# Supplementary material for: Co-developing a comprehensive disease policy model with stakeholders: The case of malaria during pregnancy
Source: PLOS Glob Public Health. 2025 May 7;5(5):e0003775. doi: 10.1371/journal.pgph.0003775 (PMC12057981; doi:10.1371/journal.pgph.0003775)
Supplement: S1 Text — (DOCX) [file pgph.0003775.s001.docx]

**S1 Text :** Detailed methods and results

Paper title: **Co-developing a comprehensive disease policy model with stakeholders: the case of malaria during pregnancy**

Authors: Authors: Silke Fernandes*; Andrew Briggs; Kara Hanson

silke.fernandes@lshtm.ac.uk

Contents

[Methods 2](#_Toc174123105)

[Results 5](#_Toc174123106)

[Figures 10](#_Toc174123107)

[Fig A: Conceptual model: Draft 2- Delphi consultation post round 1 10](#_Toc174123108)

[Fig B: Conceptual model: Draft 3- Delphi consultation post round 2 12](#_Toc174123109)

[References 14](#_Toc174123110)

# Methods

The different stages and methods in this study are illustrated in Fig 1 (main paper). During an initial preparation stage, a steering group was convened. Its task was to short-list experts to be approached to be part of a Delphi panel and to advise on the preparation of a first draft of the conceptual model and questionnaire based on their understanding of the literature, ongoing research and natural history of malaria in pregnancy. The subsequent Delphi consultation stage relied on the knowledge and expertise of the Delphi panel to refine the draft model using an a priori undetermined number of rounds of consultations until consensus in most questions was reached. The task of the experts was to rate the importance of a set of outcomes, to suggest additional outcomes and their relationships, and to identify suitable stratifiers. In a final stage, two consensus meetings were held to vote on outstanding questions, reach full consensus amongst the experts and finalize the model.

**Stage one: Preparation**

At the start of the study, members of a small steering group were identified by the core research team (first and last author), consisting of one expert in the clinical epidemiology of malaria during pregnancy, one expert in health economics with experience in conceptual models and the core research team, both experts in health economics of malaria during pregnancy. The steering group used their experience, knowledge of literature, ongoing research and additional literature searches in Pubmed to advise on the first draft of the conceptual model for the cost-effectiveness analysis of malaria in pregnancy. This first draft was used as a starting point for the Delphi consultation process. A survey based on the first draft of the conceptual model was developed and piloted using two researchers who were neither on the steering group nor Delphi panel. Potential candidates for the Delphi panel were purposively selected and approached by email for their varied expertise, experience, knowledge of the literature and current research in malaria during pregnancy. The inclusion criteria for experts in this study were: specialist expertise in malaria during pregnancy with technical expertise in clinical research, epidemiology, health economics, disease modelling or implementation research. We paid particular attention to having a well-balanced panel with experts representing both maternal and child health, early and later exposure to malaria during pregnancy and various endemicity contexts.

We aimed to include 8-10 experts in our Delphi panel, a group size shown to be effective and reliable for the Delphi method [1, 2]. Experts received no incentive or financial reimbursement for their time participating in this study.

**Stage two: Delphi consultation**

Two rounds of Delphi consultations were administered online.

*Delphi Round one*

The first round was sent via a Google form online survey (see S2 Text) on 21 September 2022 together with a draft of the conceptual model (Fig 2 and Fig 3 in main paper). The first part of the survey included an information sheet and consent form with some general background questions. Subsequent questions aimed to establish consensus on outcomes to be included in the model, relationships between outcomes and to identify the most relevant stratifiers to be included in the next version of the conceptual model. The Delphi panel members were asked to perform the following six tasks: 1) Assess importance of the outcomes in the model, 2) suggest additional outcomes that were missing, 3) evaluate the accuracy of relationships between outcomes, 4) suggest additional relationships between outcomes that were missing 5) order stratifiers (or effect modifiers) for subgroup analysis by importance and 6) suggest any additional stratifiers that were missing. We used nominal and ordinal categorical response options. For example when asking about outcomes to be included in the model, panellists could choose option 1 to 6 with categories 1 and 2 considered “not important for inclusion”, categories 3 and 4 “important but not critical” and 5 and 6 ”critical to include”. In addition panellists could select “unsure” as a response option. For each categorical question candidates were given the option to provide free-text comments to support their response. Panellists were also asked free text questions on additional outcomes, relationships or other factors to be added to the model as well as on their viewpoint of certain aspects of the model, such as the components and visual presentation of low birth weight. Categorical questions were analysed using simple descriptive statistics. Free text responses were explored using a simple thematic analysis, coding them manually into themes [3]. Consensus was set *a priori* at 70% agreement, which is consistent with previous Delphi studies reported in the literature [4, 5]. The response “unsure” was excluded from percentage estimations of categorical questions. Where the panel did not reach at least 70% agreement to include, exclude or modify an outcome, relationship or other factor questioned, we examined the comments and proposed suitable follow up questions for round two and kept the outcome in the model. Any additional outcomes, relationships and other factors suggested by experts on the panel were explored using a literature search and if supported by evidence added to the questions in the next round of the Delphi consultation. One researcher (SF) analysed the responses during a four week period. Based on the responses by the panellists a new version of the conceptual model was drafted (Fig A).

For detail of the questionnaire used in round one, please see S2 Text.

*Delphi Round two*

Experts received a summary report (S4 Text) of the analysis from round one, ensuring anonymity was maintained, together with the new version of the conceptual model (Fig A) and the second round of the Delphi consultation online survey on 4 December 2022 (S3 Text). The round two questionnaire was based on the new version of the conceptual model and built upon the panellist responses in round one. Questions sought to clarify any discrepancies from round one and to continue with the same six tasks described above. All twelve experts who gave consent to the study completed their round two questionnaire. At the end of round two, the analysis, summary report of round two (S5 Text) and redrafting of the conceptual model (Fig B) was repeated.

**Stage three: Consensus meeting**

Two online meetings for experts in different time zones were held to present the findings of the second Delphi round as well as to discuss and vote on any remaining aspects of the model where consensus had not been reached during stage two. All Delphi panel and steering group members were invited, however only Delphi panellists were permitted to vote. The aim of this meeting was to finalize outcomes, relationships and other factors to be included in the final model. Questions where consensus had been reached in stage two were not discussed during the consensus meeting. The conceptual model was finalized by the first author following the consensus meeting.

# Results

**Stage one: Preparation**

The first draft of the conceptual model developed by the steering group included outcomes for the mother and the child and relationships between different outcomes (Fig 2 and Fig 3 in the main paper). Gravidity, timing of exposure to *P.falciparum* (i.e. first, second or third trimester) and HIV status were selected as the most relevant stratifiers (effect modifiers) for subpopulation analysis to feed into the first round of the questionnaire. The steering group identified 17 experts as potential candidates for the Delphi panel. All 17 experts were approached to participate in the study, of which twelve agreed (71%). Reasons to decline were “no reply” (N=3) and “lack of time” (N=2). One member of the steering group was also on the Delphi panel (Prof. Feiko ter Kuile).

Individual outcomes were divided into outcomes related to morbidity and mortality. To visually differentiate outcomes with short-, mid- and long-term effects, a colour code was added to each outcome to indicate the timing of each health outcome.

**Stage two: Delphi consultation**

*Round one:*

The twelve experts who consented to the study were affiliated with a wide range of institutions located in Africa (N=3), Asia (N=1), North America (N=3), Australia (N=2), Europe (N=2) and internationally (N=1). All experts agreed to be named as a member of the Delphi panel in a subsequent publication. The average years of experience working in malaria in pregnancy was 17.9 years (SD=8.5) with a range of 8-34 years, with exception of one expert without explicit experience in malaria during pregnancy, but over 15 years in the economics of malaria.

Most maternal and child outcomes as well as relationships between them presented in draft 1 (Fig 2 and Fig 3, main paper) achieved 70% consensus to be important and critical for the model. Three outcomes below the 70% consensus to be important and critical for the model (long-term neurological sequelae, fetal anaemia, congenital malaria), also did not achieve consensus to be dropped and therefore remained in the model.

On recommendation of panel members, "severe disease” and “serious complications” were combined into a single outcome of “severe malaria” as a number of experts pointed out the difficulty in differentiating between these two outcomes. A new relationship from “maternal anaemia” to “severe disease” was added to demonstrate the important contribution of maternal anaemia to severe malaria [6]. The relationship between “maternal anaemia” and “clinical malaria” was changed to be bi-directional based on feedback from the Delphi panel and was explored further in the second round. For completeness, “miscarriage/stillbirth” was relabelled to also include the death of the baby in utero if the mother dies (“death in utero after maternal death”).

All experts agreed that “low birth weight” should be separated into “intrauterine growth restriction” and “preterm birth”, with five experts suggesting the addition of “small for gestational age”. The label of “short-, medium and long-term morbidities” was replaced with neonatal, infant, <5, older child and adult morbidities and an arrow leading to mortalities was drawn.

Additional maternal outcomes to be incorporated into the next draft of the model were “asymptomatic parasitaemia”, “placental malaria” and “hypertension disorders of pregnancy”. Both asymptomatic parasitaemia and placental malaria would not be considered relevant outcomes in an economic analysis, but important proxy outcomes for epidemiologists and disease modellers. Therefore, “placental malaria” was not added as a separate outcome, but combined with “asymptomatic parasitaemia” and “clinical malaria”. No additional child outcomes were suggested by the experts.

Additional suggestions made by experts but not included in the next draft after careful consideration of the evidence and scope of the model were “pre-conception exposure to malaria” (suggested by one expert, considered out of scope of the model) and “postpartum maternal infections due to low immunity caused by pregnancy and malaria infection” (suggested by one expert, no clear evidence identified).

Experts were asked to rate three stratifiers or effect modifiers (gravidity, HIV status, timing of exposure) by importance, however, results were not conclusive and required further exploration. Three additional stratifiers were suggested more than once: Transmission intensity, nutritional status of mother, infant gender.

All changes or uncertainties were explored in the next round of the Delphi consultation. The model was redesigned graphically incorporating suggestions made by different experts, for example the insertion of symbols to assist readers with colour blindness in addition to the colour coding representing the timing of the health effects (see Fig A for the draft of the model following Delphi consultation round one).

S4 Text contains a summary report of the round one analysis.

*Round two:*

All experts from round one also participated in round two. The questionnaire in round two contained more free text questions than round one and was analysed accordingly.

Experts reviewed the inclusion and presentation of additional outcomes in the model. They reached consensus regarding the inclusion of asymptomatic parasitaemia (100%, 12/12 agreed) and placental malaria (75%, 9/12 agreed) and their associated relationships. Whether to include or exclude “hypertension disorder of pregnancy” was unclear and had to be explored further during the consensus meeting. While all relationships associated with “hypertension disorder of pregnancy” were judged to be correct, it appeared that there was a difference in opinion regarding its importance and relevance amongst experts working in low- versus high endemicity settings.

The majority concurred on the suggested presentation of severe malaria (83%, 10/12), which now also includes severe anaemia. Similarly, most experts (83%, 10/12) agreed with the presentation of preterm birth, intrauterine growth restriction and small for gestational age. The suggested bi-directional relationship between clinical malaria and maternal anaemia caused some confusion amongst experts and had to be clarified during the consensus meeting.

In addition, other questions and comments in round two aimed to improve the wording or design of certain aspects of the model. For example, the addition of symbols to represent the timing of outcomes in addition to the colour coding was found useful by 83% (10/12) of experts. “Long term neurological sequelae” was extended to “long term neurological sequelae and other sequelae”, as a number of experts pointed out that non-neurological sequelae, for example chronic kidney disease need to be represented [7].

Experts raised a few additional issues related to coherent presentation of the model and accurate outcome names, which were incorporated into the agenda for the consensus meeting.

Lastly, experts were asked to vote for the two most important stratifiers (effect modifiers) for subpopulation analysis leading to the following ranking from most to least important with the number of votes in brackets: gravidity (10), transmission intensity (8), timing of exposure of P.falciparum (3) and HIV status (2) (one expert voted only for one).

S5 Text contains a summary report of the round two analysis and Fig B shows the draft of the model following Delphi consultation round 2.

**Stage three: Online consensus meeting**

The two one-hour consensus meetings, held on 31^st^ August and 8^th^ of September 2023, were attended by 9 out of 12 (75%) members of the Delphi panel as well as three members of the steering group. The three overarching topics for discussion were 1) hypertension disorders of pregnancy, 2) the relationship between anaemia and clinical malaria and 3) childhood morbidities. In both consensus meetings, experts were respectful and considerate towards each other, offering their opinion wisely and engaging humbly with other expert’s views.

After engaged discussions, all attending experts (9/9, 100%) voted to keep “hypertension disorder of pregnancy” in the model. A small majority (5/9, 56%) voted against splitting “hypertension disorder of pregnancy” further into “hypertension”, “pre-eclampsia” and “eclampsia”, however they decided to add “long-term effects of hypertension disorders of pregnancy” as a further outcome to include long-term sequelae such as stroke or mental health disorders. Even though the pre-defined 70% threshold for consensus wasn’t reached, the majority decision made by the experts was followed.

The suggested bi-directional relationship between “clinical malaria” and “anaemia” was debated, particularly during the first meeting. One expert pointed out the importance of differentiating between an association and a causal pathway from “anaemia” to “clinical malaria”. During the final vote, experts settled on removing the bi-directional relationship and only retaining the arrow from “clinical malaria” to anaemia (8/9, 89%), as a causal pathway from anaemia to clinical malaria was considered unlikely.

A number of less contentious issues related to child morbidities were voted on during the last part of each meeting. The majority of experts agreed that additional arrows from i) “Child- exposure to *P.falciparum*”, ii) congenital malaria and iii) fetal anaemia to other morbidities were needed. The outcome “neurocognitive development impairment in <5” was extended to “physical and neurocognitive development impairment in <5” and “modified incidence of malaria in <5” was relabelled to “increased incidence of malaria in <5”. Lastly, small changes to the graphical presentation, for example changing the shape of “other morbidities” were agreed.

Table 1 (main paper) contains a summary of all changes made between different models.

Experts expressed the importance of adapting economic models to context and allowing the flexibility for them to evolve over time as more granular data become available. They also felt that in addition to developing a conceptual model of malaria during pregnancy to be used in future cost-effectiveness analysis, the work had helped to identify a number of areas where data are limited and that it will be important to share these with the research community. The final model is shown in Fig 4 (main paper), in which both child and maternal figures are combined, as suggested during the consensus meeting.

# Figures

## Fig A: Conceptual model: Draft 2- Delphi consultation post round 1

S1a) Maternal outcomes


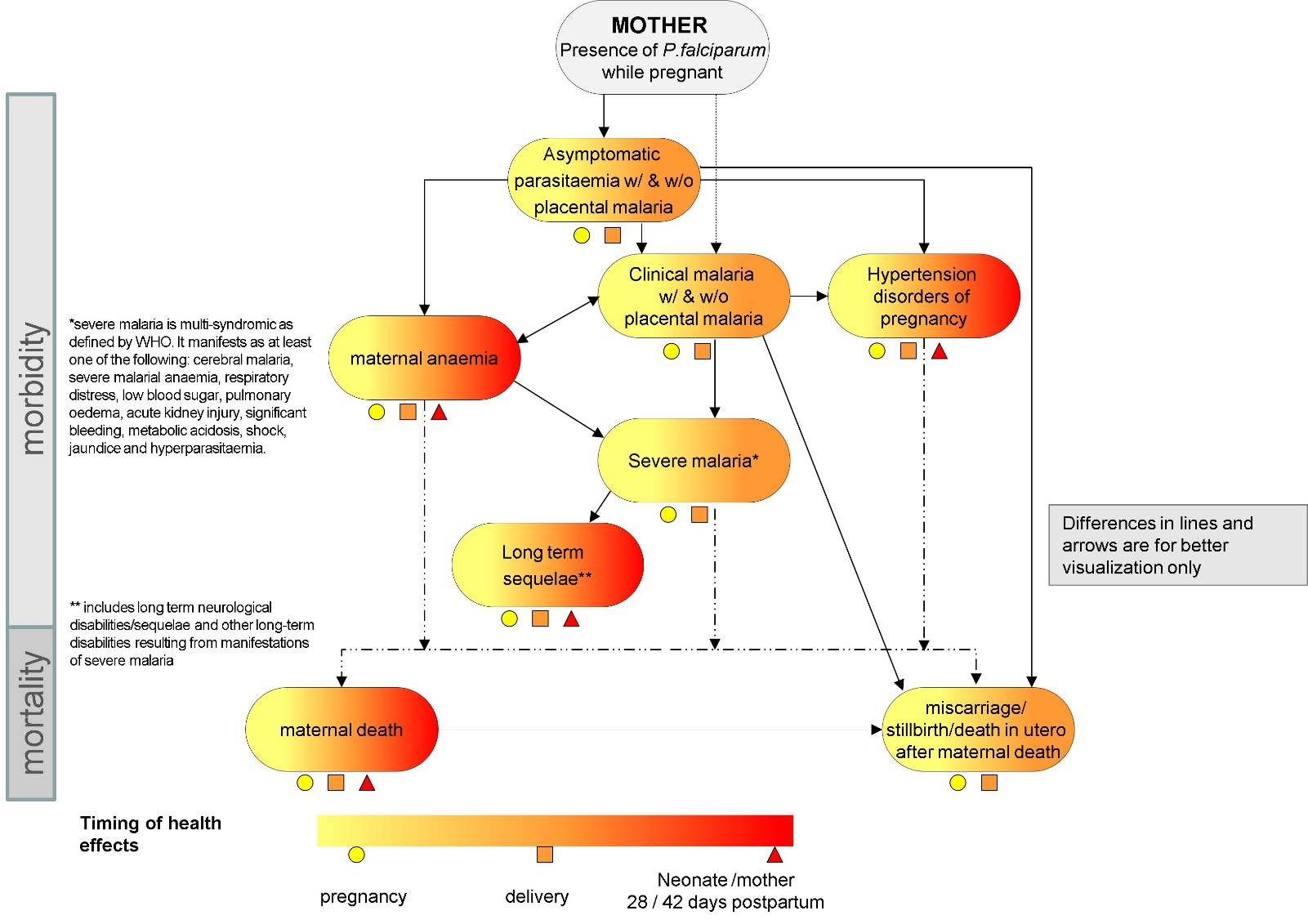


S1b) Child outcomes
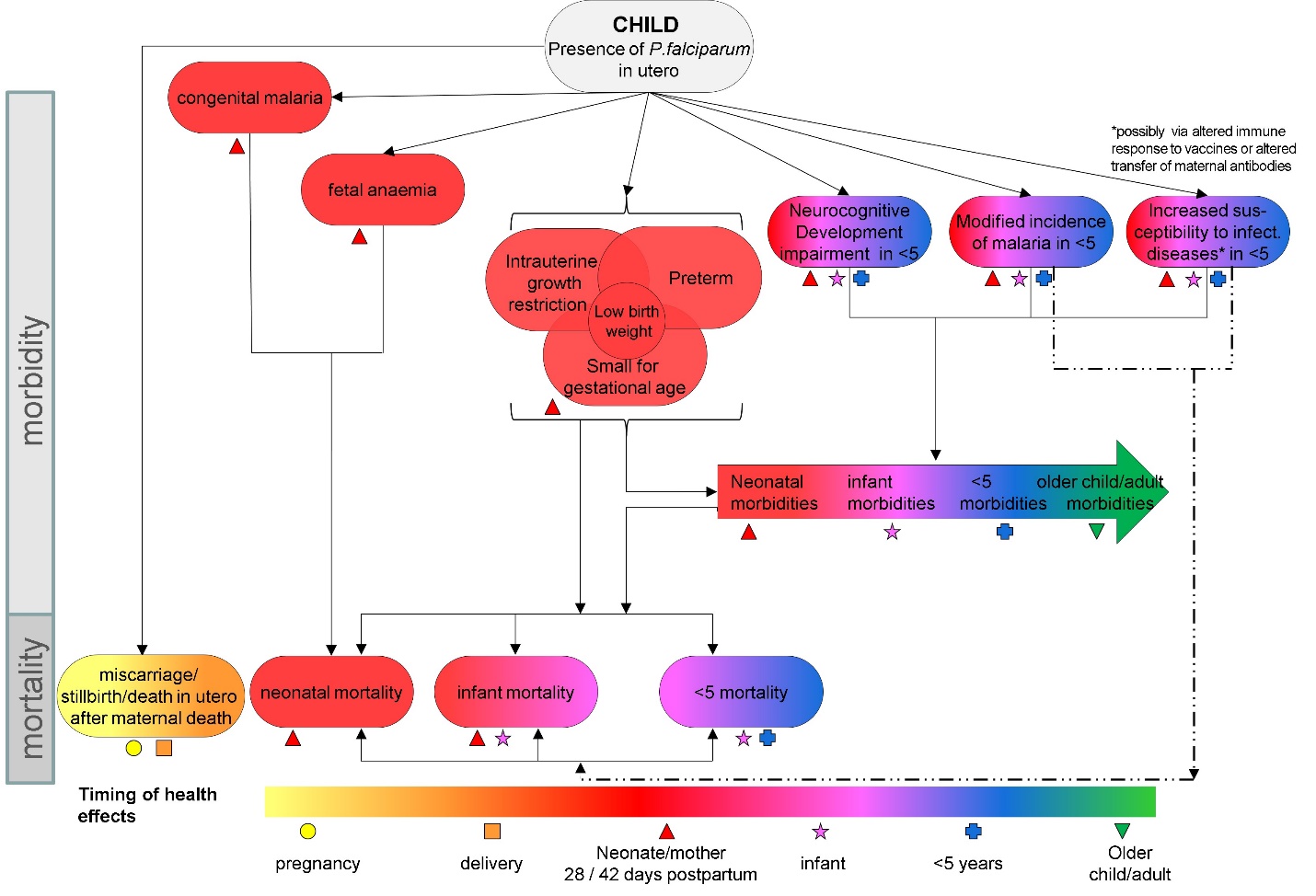


**Fig A** shows the maternal and child outcomes included in the second draft of the conceptual model developed based on the feedback received during the first round of the Delphi consultation with a panel of 12 experts. Abbreviations: w/=with; w/o=without; WHO=World Health Organization

## Fig B: Conceptual model: Draft 3- Delphi consultation post round 2

S2a) Maternal outcomes


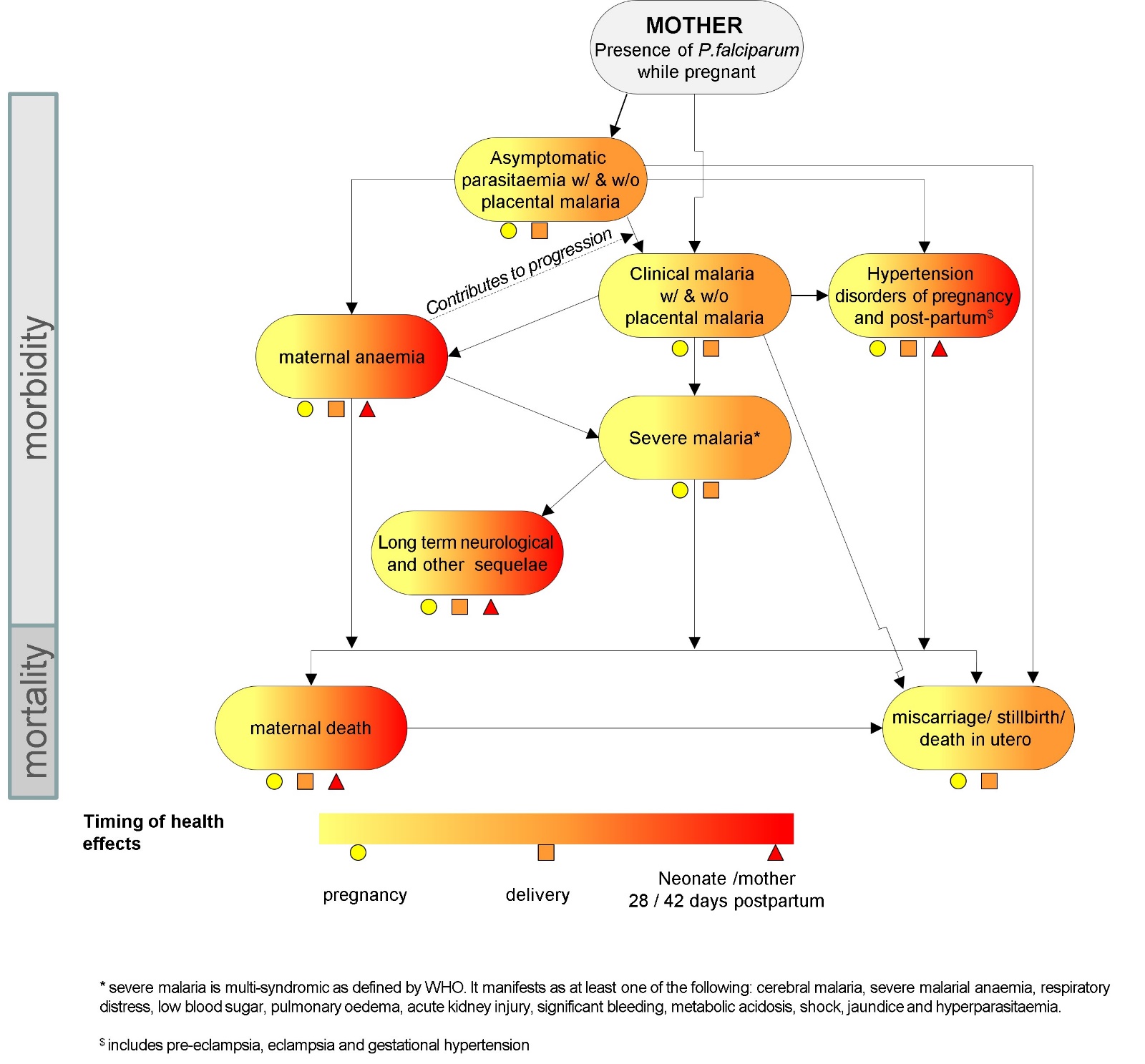


S2b) Child outcomes


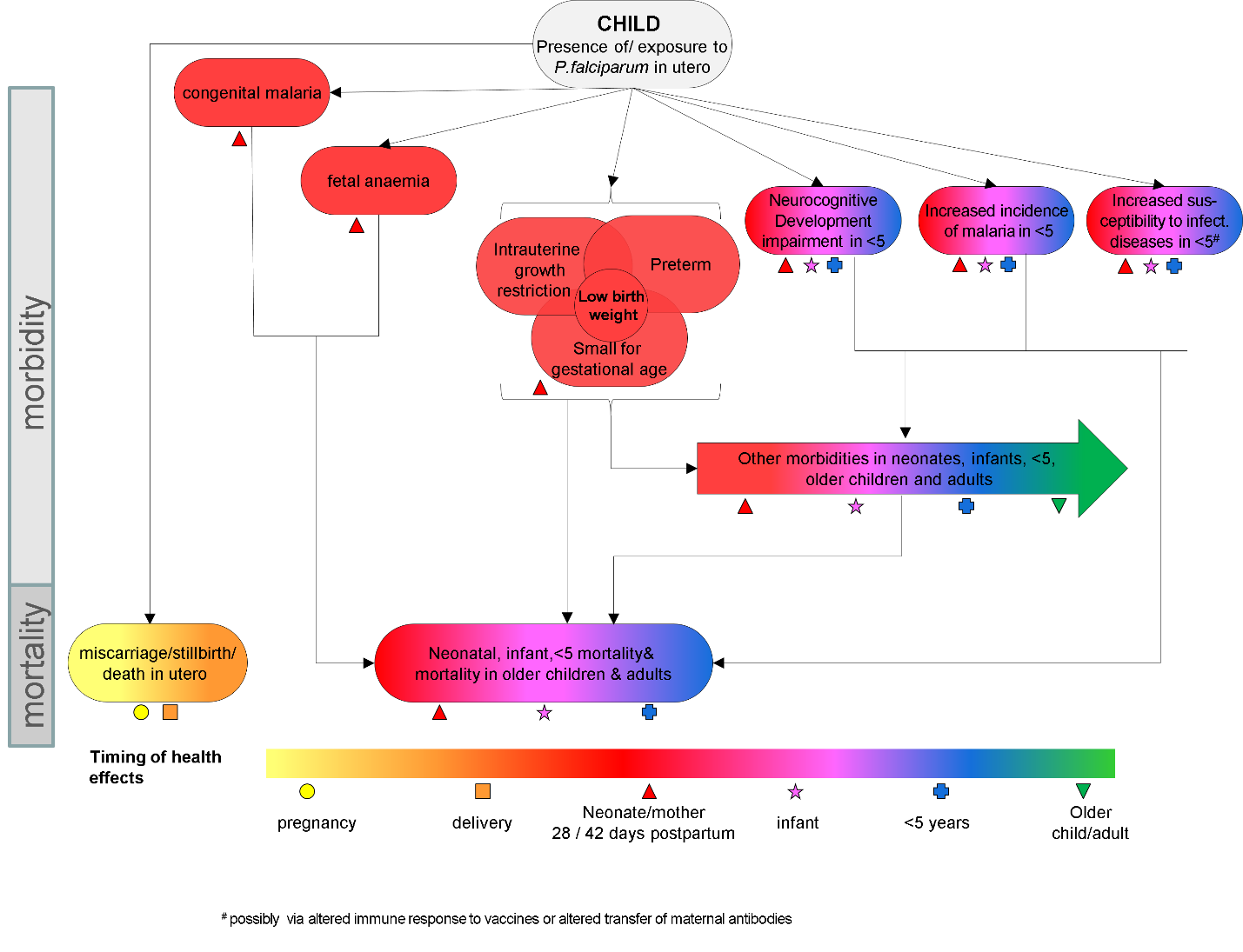


**Fig B** shows the maternal and child outcomes included in the third draft of the conceptual model developed based on the feedback received during the second round of the Delphi consultation with a panel of 12 experts. Abbreviations: w/=with; w/o=without; WHO=World Health Organization

# References

1. Dalkey NC. The Delphi Method: An Experimental Study of Group Opinion. Santa Monica, CA: RAND Corporation; 1969.

2. Jones J, Hunter D. Qualitative Research: Consensus methods for medical and health services research. BMJ. 1995;311(7001):376. doi: 10.1136/bmj.311.7001.376.

3. Braun V, Clarke V. Using thematic analysis in psychology. Qualitative Research in Psychology. 2006;3:77-101. doi: 10.1191/1478088706qp063oa.

4. Woodcock T, Adeleke Y, Goeschel C, Pronovost P, Dixon-Woods M. A modified Delphi study to identify the features of high quality measurement plans for healthcare improvement projects. BMC Med Res Methodol. 2020;20(1):8. Epub 20200114. doi: 10.1186/s12874-019-0886-6. PubMed PMID: 31937262; PubMed Central PMCID: PMCPMC6961316.

5. Tabberer M, Gonzalez-McQuire S, Muellerova H, Briggs AH, Rutten-van Molken M, Chambers M, Lomas DA. Development of a Conceptual Model of Disease Progression for Use in Economic Modeling of Chronic Obstructive Pulmonary Disease. Medical decision making : an international journal of the Society for Medical Decision Making. 2017;37(4):440-52. doi: 10.1177/0272989X16662009. PubMed PMID: 27486218.

6. Severe Malaria Observatory. Severe Malaria Criteria, Features & Definition [15/9/23]. Available from: <https://www.severemalaria.org/severe-malaria/severe-malaria-criteria-features-definition>.

7. Brown DD, Solomon S, Lerner D, Del Rio M. Malaria and acute kidney injury. Pediatr Nephrol. 2020;35(4):603-8. Epub 20190131. doi: 10.1007/s00467-018-4191-0. PubMed PMID: 30706124.
