# Supplementary material for: Co-developing a comprehensive disease policy model with stakeholders: The case of malaria during pregnancy
Source: PLOS Glob Public Health. 2025 May 7;5(5):e0003775. doi: 10.1371/journal.pgph.0003775 (PMC12057981; doi:10.1371/journal.pgph.0003775)
Supplement: S2 Text — (PDF) [file pgph.0003775.s002.pdf]

CEA conceptual model of malaria in pregnancy prevention

Delphi study consultation round 1 survey

* Indicates required question

Information sheet page 1

LONDON SCHOOL of HYGIENE &TROPICAL MEDICINE

Information sheet

We would like to invite you to take part as an expert in a Delphi consensus study. Before you decide whether or not you are willing to take part, we would like you to read this information sheet carefully.

What is the study title?

Development of a Conceptual Model for use in cost-effectiveness modelling of Malaria in Pregnancy Prevention.

What is a Delphi study?

The Delphi technique seeks to obtain consensus on the opinions of panel members, through a series of structured questionnaires administered in an iterative multi-round process.

What is the purpose of this study?

The aim of this consensus study is to develop with a group of experts a model for use in cost-effectiveness analysis of malaria in pregnancy prevention.

Why have I been invited to take part?

As an established expert in this field we are keen to have you on our panel of experts to gain your views about which outcomes, costs, associations/relationship and other factors may be important in this model.

What will I do?

In each round, we will send you a draft model and will ask you to fill in an online survey, in which you will be asked to review and rate possible outcomes, associations/ relationships, costs and other factors shown in the model draft. You will also have the opportunity to suggest additional outcomes, associations/ relationships, costs and other factors that were not included in the draft model. It is envisaged that each round should take approximately 45-60 min to complete and we anticipate 2-3 rounds until consensus is reached.

In order to allow timely conclusion of the study we would respectfully request a response time of 1 week for completion of each round. We aim to analyse each round within 1 week of receipt of all responses.

After the final round, we would like to invite you to a consensus meeting, **provisionally scheduled for the 20th of October 2022 at 2 p.m. BST.** We would kindly ask all panelists to try to attend this meeting, which will be an opportunity to finalize the model.

What are the benefits and risks?

We don't anticipate any direct benefits nor risks or discomfort to you by agreeing to participate in the study, however your contribution will help to develop a robust model of malaria during pregnancy that can be used in future cost-effectiveness analyses.

As we will be using the expertise of the panel's members to give credibility to the findings of the Delphi study, participants cannot be anonymous, however your answers will be anonymised.

Will the information collected be confidential?

If you participate in the research, we will do everything possible to protect the confidentiality of your responses.

In addition we will also ask you to consent to be named as a participant of the Delphi panel in any publications.

Information sheet page 2

Future research:

Your data won't be used or shared for any future research studies.

Funding source:

Participants in the Delphi panel will not be paid. Silke Fernandes received financial support from the EDCTP2 programme and the MRC/DFID/ Wellcome Trust's Joint Global Health Trials (JGHT) scheme to the Liverpool School of Tropical Medicine (Grant no TRIA-2015-1076-IMPROVE and Grant no TRIA-2015-1076b-IMPROVE-2) to conduct the Cost-effectiveness analysis of the Improve 1 and Improve 2 study. The conceptual framework forms part of Silke Fernandes' PhD.

Data storage and access:

Data will be stored for 10 years on a password-protected, double-firewalled https server at LSHTM in London that provides 2048-bit encryption. It is backed up on a daily basis to a secure off-site location by LSHTM's IT Services. Data can be accessed by the researchers to analyse the study and by agencies that enforce legal and ethical guidelines, such as the Institutional Review Board (IRB) at LSHTM.

What happens if I don't want to participate?

You are free to decide whether you wish to participate. Participation is voluntary. You are also free to change your mind and stop participating at any time. If you decide to stop your participation we will ask you whether you are happy for us to use the data we have already collected from you and we will honour your decision.

If you have any further questions or concerns

If you have questions about this study, please contact Silke Fernandes at the London School of Hygiene & Tropical Medicine, Keppel Street, London, UK, WC1E 7HT by telephone +44(0)7515436924 or email silke.fernandes@lshtm.ac.uk.

If you have any questions or concerns about your rights as a research participant or if you want to discuss a problem, get information or offer input by talking to someone who is not part of the research team you may contact:
LSHTM Ethics Committee, Keppel Street, London, UK, WC1E 7HT; Daytime telephone number: +44 (0) 20 7636 8636

This proposal has been reviewed and approved by the London School of Hygiene & Tropical Medicine Ethics Committee. This committee makes sure that research participants are protected from harm.

IRB: London School of Hygiene and Tropical Medicine

IRB LSHTM Ethics reference number: 27361

IRB Approval Date: 12 July 2022

Consent Form

Title:
Development of a Conceptual Model using a consensus study for use in Economic Evaluation Modelling of Malaria in Pregnancy Prevention

Researchers:
Silke Fernandes, PhD student, Department of Global Health and Development, LSHTM
Kara Hanson, Professor, Faculty of Public Health and Policy, LSHTM

By agreeing to be part of this study, you agree to all the following:

- I have read the information sheet that explains the reason for the study, and the procedures that I will be asked to participate in.
- I understand that I am free to choose whether or not I wish to participate, and that no pressure will be put on me to participate.
- All the questions I had about this study have been answered.
- I understand that I can request to stop participating in this study at any time, and that it will stop immediately upon my request.
- I have been given sufficient time to consider whether to take part in this research.

1. 1. Do you agree to be on this Delphi study panel? *

Mark only one oval.

◯ Yes *Skip to question 5*
◯ No *Skip to section 9 (Participation Declined)*

2. 2. Full name *

____

3. 3. Email address *

____

4. 4. As the experience and reputation of the experts on the panel will contribute to the credibility of the results, we would like to ask you if you you agree to be named as a member of the Delphi Panel in future publications and documents (such as the PhD thesis). Your response will not affect your participation in this research. *

Mark only one oval.

- ◯ Yes, I agree to be named as a member of the Delphi Panel
- ◯ No, I do not agree to be named as a member of the Delphi Panel
  Skip to section 9 (Participation Declined)

General information section

5. B1. Area of expertise? (please tick all the apply) *

Tick all that apply.

- ☐ Epidemiology
- ☐ Clinical
- ☐ Disease modelling
- ☐ Social sciences
- ☐ Implementation research
- ☐ Health economics
- ☐ pathogenesis
- ☐ Clinical trials
- ☐ Other: ______________________

6. B2. Years of experience working in malaria in pregnancy? *

____

7. B3. What is your main institutional affiliation? *

____

Introduction

You have been sent a draft model in a separate email (it can also be downloaded here: [Conceptual model round 1](#)). This model was developed by a small panel of experts in malaria in pregnancy and health economics. The aim of this study is to develop this draft model into a final model by using the expertise of the panel members. The final model can then be used in future cost effectiveness analysis of malaria in pregnancy interventions. To show our appreciation of your time and effort - provided we received consent from you - we will acknowledge your contribution to this panel in all future publications.

When reviewing this draft model and answering the questions in this survey, we would like you to **initially focus on the relevance and importance** of each parameter, always ensuring that there is sufficient evidence to support the inclusion of the parameter in the final model. Parameters can include outcomes, relationships, costs, stratifiers and other factors such as time horizon and analysis perspective. **At a later stage we will ask you to consider data availability.**

Thank you so much again for agreeing to take part in this work, your expertise is invaluable.

Maternal health outcomes in the model

In this section we will review the maternal outcomes and the relationships included in the draft model. Miscarriage and stillbirth have been included here under maternal outcomes, even though they are outcomes relevant to both the mother and the child.

<u>Health outcomes in the mother</u>

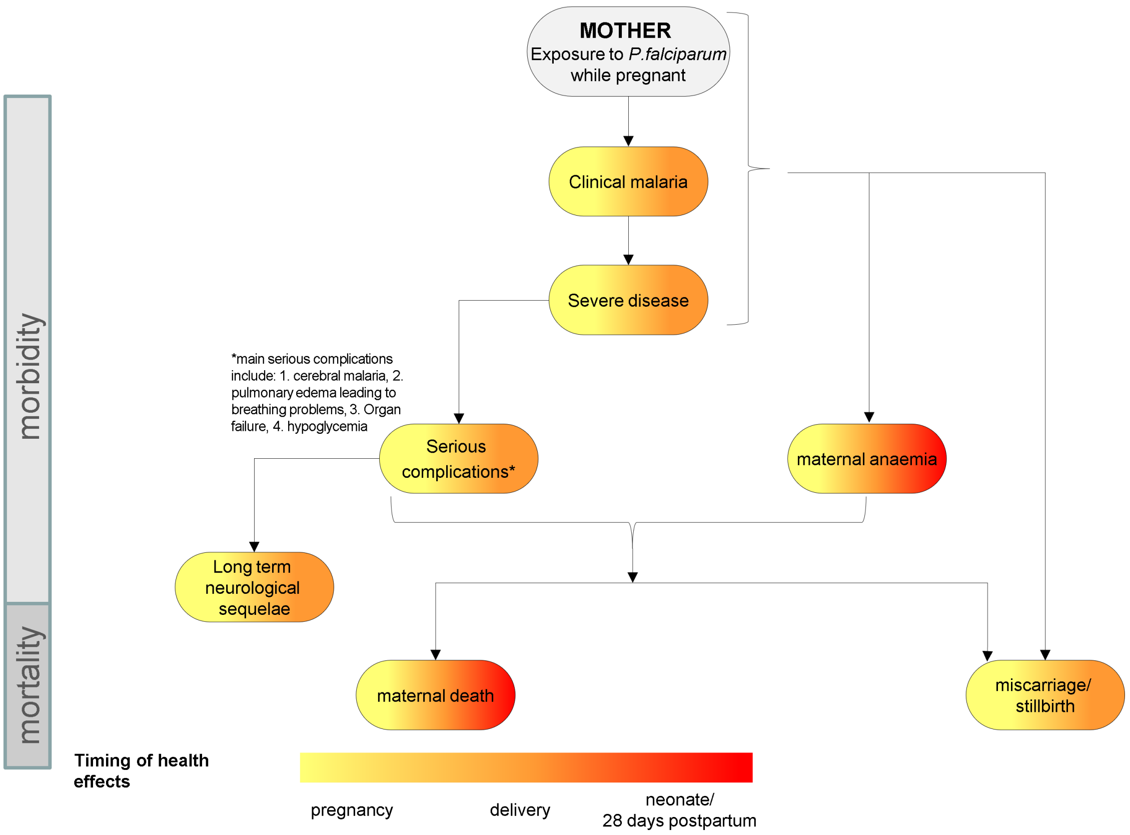

8. M1. Please could you carefully review the maternal outcomes shown in the picture above (or on page three here: Conceptual model round 1) and indicate for the following outcomes which ones should be included in the model by rating their importance for inclusion on a scale 1-6. *

We consider:
1-2 as not important for inclusion
3-4 as important but not critical
5-6 as critical to include

Mark only one oval per row.

	1	2	3	4	5	6	unsure
**Clinical malaria**	⬭	⬭	⬭	⬭	⬭	⬭	⬭
**Severe disease (malaria)**	⬭	⬭	⬭	⬭	⬭	⬭	⬭
**Maternal anaemia**	⬭	⬭	⬭	⬭	⬭	⬭	⬭
**Serious complications**	⬭	⬭	⬭	⬭	⬭	⬭	⬭
**Long term neurological sequelae**	⬭	⬭	⬭	⬭	⬭	⬭	⬭
**Maternal death**	⬭	⬭	⬭	⬭	⬭	⬭	⬭
**Miscarriage**	⬭	⬭	⬭	⬭	⬭	⬭	⬭
**Stillbirth**	⬭	⬭	⬭	⬭	⬭	⬭	⬭

9. M2. If you have any comments regarding the previous question, please leave them here:

____

____

____

____

____

10. M3. Maternal outcome "serious complications" following "severe disease": Do you think serious complications can be combined in the model as shown above or on page 3 of the model draft or should each of the four listed complications be shown individually? Please give a brief answer with justification. *

11. M4. Would you like to suggest any other maternal outcomes to be included in the model? If possible, please support your suggestion with evidence.

12. M5. Before moving onto relationships between maternal outcomes in the model, do you have any other comments regarding the maternal outcomes?

13. M6. For the **relationships** (indicated by the arrows) between maternal outcomes shown above (or on page 3 of the model draft), please indicate whether you think they are correct or incorrect (indicating what correction is needed) *

Mark only one oval per row.

	correct	incorrect, remove	incorrect, change direction	incorrect, make bi-directional	incorrect, other (specify)	unsure
**Exposure to P. falciparum –> clinical malaria**	⬭	⬭	⬭	⬭	⬭	⬭
**Exposure to P.falciparum –> miscarriage/stillbirth**	⬭	⬭	⬭	⬭	⬭	⬭
**Exposure to P.falciparum –> maternal anaemia**	⬭	⬭	⬭	⬭	⬭	⬭
**Clinical malaria –> severe disease**	⬭	⬭	⬭	⬭	⬭	⬭
**Clinical malaria –> miscarriage/stillbirth**	⬭	⬭	⬭	⬭	⬭	⬭
**Clinical malaria –> maternal anaemia**	⬭	⬭	⬭	⬭	⬭	⬭
**Severe disease –> serious complications**	⬭	⬭	⬭	⬭	⬭	⬭
**Severe disease –> miscarriage/stillbirth**	⬭	⬭	⬭	⬭	⬭	⬭
**Severe disease –> maternal anaemia**	⬭	⬭	⬭	⬭	⬭	⬭
**Serious complications –> long-term neurological sequelae**	⬭	⬭	⬭	⬭	⬭	⬭
**Serious complications –> miscarriage/ stillbirth**	⬭	⬭	⬭	⬭	⬭	⬭
**Serious complications –> maternal death**	⬭	⬭	⬭	⬭	⬭	⬭

Maternal anaemia --> miscarriage/stillbirth						
Maternal anaemia --> maternal death						

14. M7. If you suggested "incorrect, other" for any of the relationships between maternal outcomes in the previous question, please explain here:

15. M8. If you have any comments regarding the relationships between maternal outcomes, please leave them here:

16. M9. Would you like to suggest any other relationships between maternal outcomes to be included in the model? If you suggested additional maternal outcomes previously, please add any relevant relationships here. If possible, please support your suggestion with evidence.

17. M10. Before moving onto child outcomes in the model, do you have any other comments regarding the relationships between maternal outcomes?

Child outcomes in the model

In this section we will review the child outcomes and the relationships included in the draft model. Miscarriage/ stillbirth has already been included under maternal outcomes and will not be asked about again here.

Outcomes in the child

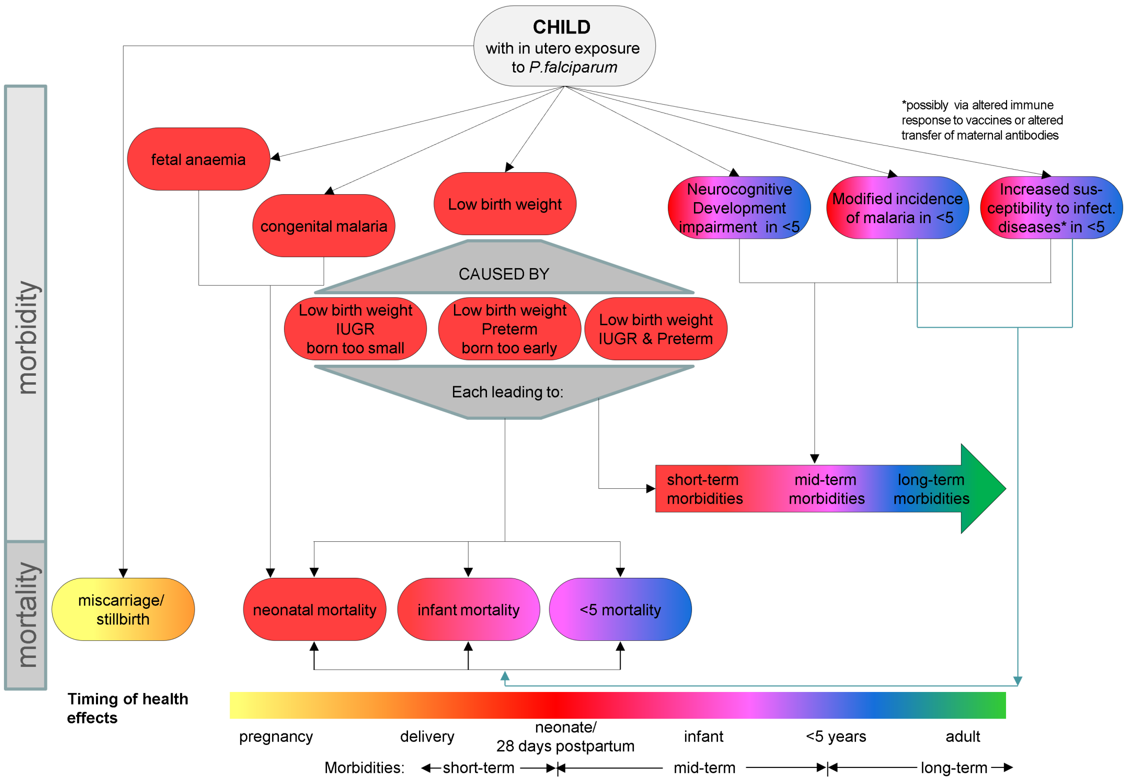

18. C1. Child outcomes in the model: Please could you carefully review the child outcomes shown in the picture above (or on page 4 here: Conceptual model round 1) and indicate for the following outcomes which ones should be included in the model by rating their importance for inclusion on a scale 1-6 *

We consider:
1-2 as not important for inclusion
3-4 as important but not critical
5-6 as critical to include

Mark only one oval per row.

	1	2	3	4	5	6	unsure
**Fetal anaemia**	◯	◯	◯	◯	◯	◯	◯
**Congenital malaria**	◯	◯	◯	◯	◯	◯	◯
**Low birth weight**	◯	◯	◯	◯	◯	◯	◯
**Neurocognitive development impairment in <5**	◯	◯	◯	◯	◯	◯	◯
**Modified incidence of malaria in <5**	◯	◯	◯	◯	◯	◯	◯
**Increased susceptibility to infectious disease in <5**	◯	◯	◯	◯	◯	◯	◯
**short/mid/long-term morbidities**	◯	◯	◯	◯	◯	◯	◯
**Neonatal mortality**	◯	◯	◯	◯	◯	◯	◯
**Infant mortality**	◯	◯	◯	◯	◯	◯	◯
**<5 mortality**	◯	◯	◯	◯	◯	◯	◯

19. C2. If you have any comments regarding the previous question, please leave them here:

20. C3. Child outcome "low birth weight": Do you think low birth weight should be separated into the three groups (IUGR, preterm, IUGR&preterm) as shown above or on page 4 of the model draft? Please give a brief answer with justification. *

21. C4. Would you like to suggest any other child outcomes to be included in the model? If possible, please support your suggestion with evidence.

22. C5. Before moving onto relationships between child outcomes in the model, do you have any other comments regarding the child outcomes?

23. C6. For the **relationships** (indicated by the arrows) between child outcomes shown above or on page 4 of the model draft, please indicate whether you think they are correct or incorrect (indicating what correction is needed) *

Mark only one oval per row.

	correct	incorrect, remove	incorrect, change direction	incorrect, make bi-directional	incorrect, other (specify)	unsure
**Exposure to P. falciparum –> fetal anaemia**						
**Exposure to P.falciparum –> congenital malaria**						
**Exposure to P.falciparum –> low birth weight**						
**Exposure to P.falciparum –> neurocognitive development impairment in <5**						
**Exposure to P.falciparum –> modified incidence of malaria in <5**						
**Exposure to P.falciparum –> increased susceptibility to infectious diseases in <5**						
**Fetal anaemia --> neonatal mortality**						
**Congenital malaria –> neonatal mortality**						
**Low birth weight –>** neonatal [illegible]						

mortality neonatal mortality						
Low birth weight --> infant mortality	◯	◯	◯	◯	◯	◯
Low birth weight --> <5 mortality	◯	◯	◯	◯	◯	◯
Low birth weight --> short/mid/long-term morbidities	◯	◯	◯	◯	◯	◯
Neurocognitive development impairment in <5 --> short/mid/long-term morbidities	◯	◯	◯	◯	◯	◯
Modified incidence of malaria in <5 --> short/mid/long-term morbidities	◯	◯	◯	◯	◯	◯
Increased susceptibility to infectious diseases in <5 --> short/mid/long-term morbidities	◯	◯	◯	◯	◯	◯
Modified incidence of malaria in <5 --> neonatal mortality	◯	◯	◯	◯	◯	◯
Modified incidence of malaria in <5 --> infant mortality	◯	◯	◯	◯	◯	◯
Modified incidence of malaria in <5 --> <5 mortality	◯	◯	◯	◯	◯	◯

malaria in <5 --> <5 mortality						
Increased susceptibility to infectious diseases in <5 --> neonatal mortality						
Increased susceptibility to infectious diseases in <5 --> infant mortality						
Increased susceptibility to infectious diseases in <5 --> <5 mortality						

24. C7. If you suggested "incorrect, other" for any of the relationships between child outcomes in the previous question, please explain here:

25. C8. If you have any comments regarding the relationships between child outcomes, please leave them here:

26. C9. Would you like to suggest any other relationships between child outcomes to be included in the model? If you suggested additional child outcomes previously, please add any relevant relationships here. If possible, please support your suggestion with evidence.

27. C10. Before moving onto stratifiers in the model, do you have any other comments regarding the relationships between child outcomes?

Stratifiers

In this section we would like to briefly review any **subpopulations** by which you think the model should be stratified.

28. S1. Subpopulations: Please could you carefully review the following parameters by which the model could be stratified. Could you please rate them by importance, 1 being the least important and 3 being the most important (please select only one for each each level of importance). *

Mark only one oval per row.

	1 (least important)	2 (average important)	3 (most important)	Not important	Not sure
**HIV status**	⬭	⬭	⬭	⬭	⬭
**Gravidity (primi, secundi and multi gravidae)**	⬭	⬭	⬭	⬭	⬭
**Timing of exposure to P.falciparum (first, second, third trimester)**	⬭	⬭	⬭	⬭	⬭

29. S2. Are there any additional parameters which you think are important and by which the analysis should be stratified? If possible, please support your suggestion with evidence.

____

____

____

____

____

Skip to section 8 (Thank you!)

Thank you!

Thank you so much for participating in this first round of our Delphi consultation. We really value your input and the time you invested in filling in this survey.

Participation Declined

You've elected not to participate in this Delphi consultation study. You can click submit or simply close your browser. Thank you.

This content is neither created nor endorsed by Google.

Google Forms
