# Supplementary material for: Co-developing a comprehensive disease policy model with stakeholders: The case of malaria during pregnancy
Source: PLOS Glob Public Health. 2025 May 7;5(5):e0003775. doi: 10.1371/journal.pgph.0003775 (PMC12057981; doi:10.1371/journal.pgph.0003775)
Supplement: S3 Text — (PDF) [file pgph.0003775.s003.pdf]

CEA conceptual model of malaria in pregnancy prevention - ROUND 2

Delphi study consultation round 2 survey

Thank you so much for your helpful feedback from round 1. It has been incredibly interesting to analyze and incorporate into the model. You will have received a summary report of the analysis, summarizing the replies and comments to the different questions asked in round 1, which you might find interesting to read.
After the feedback given by all experts, analyzing it, weighing up the different answers and comments and looking up further evidence, we have drafted a
new version of the model. Most outcomes remain in the model, with some
additional outcomes and relationships. We will explain the changes and will ask you either specific
questions or give you an opportunity to comment after explaining the change. You are absolutely free to not
comment if you are happy with the changes made or feel unable to comment. You have been sent the new version of the model in a separate email, but you can also download the new as well as the previous version here:
Version round 2: Conceptual model round 2

Previous version from round 1: Conceptual model round 1;

Thank you so much again for agreeing to take part in this work, your expertise is invaluable.

* Indicates required question

1. Please state your full name *

Maternal health outcomes in the model

In this section we will review the maternal outcomes and the relationships included in the draft model.
Any differences in lines are for visualisation purposes only.
There is no different meaning between different line styles. Similarly
differences in sizes of boxes also do not reflect importance, their size was
purely adjusted to fit the text.

Health outcomes in the mother - round 2

The main changes amongst the maternal health outcomes were:

- the addition of "asymptomatic parasitaemia" and "hypertension disorders of pregnancy" as outcomes
- changes in the way severe malaria and its consequences are presented
- relationships between some of the outcomes

Possible adverse effects in the mother associated with *P.falciparum* exposure during pregnancy

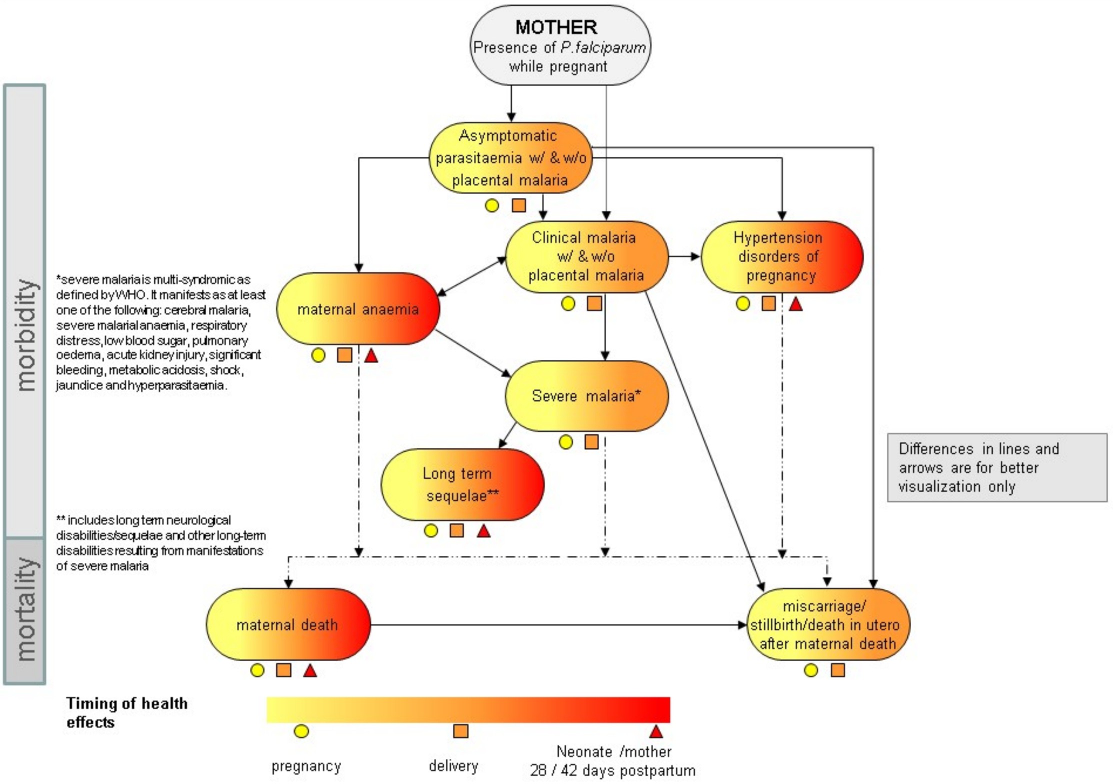

2. M1. One expert recommended to use instead of “Exposure to P.falciparum while pregnant” the term “Presence of P.falciparum while pregnant”. We felt that this is a good suggestion and have adapted this term in the model. If you would like to make any comments about this or would like to suggest something different, please do this here (feel free to leave empty if you are happy with the change):

3. M2. Many experts suggested to add asymptomatic parasitaemia (or silent infection, or sub-clinical infection etc.) as a separate outcome box. We have added it as a separate outcome box. Relationships from asymptomatic parasitaemia lead to clinical malaria, maternal anaemia, hypertension disorders of pregnancy and miscarriage/stillbirths. If you would like to make any comments regarding this new outcome and its relationships, please leave them here (feel free to leave empty if you are happy with the change):

4. M3. Many experts suggested to add placental malaria as a separate box. This has now been included with clinical malaria and asymptomatic parasitaemia as both can occur with and without placental malaria. The purpose of this model is to use it as a cost effectiveness model for malaria in pregnancy, hence outcomes will in the end be valued as DALYs and for both placental malaria and asymptomatic parasitaemia no DALY weights exist. We however felt that they show an important pathway, essential to depict in this model, even if they do not count towards the final DALY outcome. To not overcomplicate the model, we have included placental malaria not separately, but together with both asymptomatic parasitaemia and clinical malaria. Hence these two outcome boxes now read “asymptomatic parasitaemia with & without placental malaria” and “clinical malaria with & without placental malaria”. If you would like to make any comments about this, please leave them here (feel free to leave empty if you are happy with the change):

5. M4. In the previous model maternal outcomes included “severe disease”, “serious complications” and “long-term neurological sequelae”. It was suggested by several experts that the serious complications are already implied in the definitions of severe malaria and are hence not required as a separate outcome. We have now relabeled “severe disease” as “severe malaria” and removed the box “serious complications” and included a box with the WHO definitions of severe malaria, which also includes severe anaemia. If you would like to make any comments about this, please leave them here (feel free to leave empty if you are happy with the change):

6. M5. It was recommended to use “long term sequelae” instead of “long term neurological sequelae” as other organs can also suffer from long term sequelae for example chronic kidney sequelae. However, other experts pointed out that consequences of cerebral malaria are quite different from other long term disabilities caused by severe malaria. We would like to ask you which of the following you think is most suitable to describe long term sequelae of severe malaria: *

Mark only one oval.

- To use long term neurological sequelae only
- To use long term sequelae only
- To have both as separate outcome boxes
- Don't know/ unsure
- Other: ____________

7. M6. One expert commented that maternal death if before delivery can cause fetal death in utero, hence we have added a relationship from maternal death to miscarriage/stillbirth and have extended that box to include also “death in utero after maternal death”. If you would like to make any comments about this, please leave them here (feel free to leave empty if you are happy with the change):

8. M7. A number of experts suggested that **“hypertension disorders of pregnancy”** including * pre-eclampsia and eclampsia should be added to the model. Could you please indicate if hypertension disorders of pregnancy should be included in the model by rating its importance for inclusion on a scale 1-6.
We consider:
1-2 as not important for inclusion
3-4 as important but not critical
5-6 as critical to include

You will have an opportunity to comment further if needed in question M9.

Mark only one oval.

- 1
- 2
- 3
- 4
- 5
- 6
- unsure
- Other (please comment in the next question).

9. M8. For the **relationships** (indicated by the arrows) between "hypertension disorders of pregnancy" and other outcomes shown above (or on page X of the model draft), please indicate whether you think they are correct or incorrect (indicating what correction is needed) *

Mark only one oval per row.

	correct	incorrect, remove	incorrect, change direction	incorrect, make bi-directional	incorrect, other (specify in next question)	Other (specify in next question)	unsure
**Asymptomatic parasitaemia --> hypertension disorders of pregnancy**	⬭	⬭	⬭	⬭	⬭	⬭	⬭
**Clinical malaria --> hypertension disorders of pregnancy**	⬭	⬭	⬭	⬭	⬭	⬭	⬭
**Hypertension disorders of pregnancy --> miscarriage/ stillbirth**	⬭	⬭	⬭	⬭	⬭	⬭	⬭
**Hypertension disorders of pregnancy --> maternal death**	⬭	⬭	⬭	⬭	⬭	⬭	⬭

10. M9. If you have any comments regarding the added outcome "hypertension disorders of pregnancy" and its relationships to other outcomes, please leave them here:

____

____

____

____

____

11. M10. A number of experts suggested the relationship between clinical malaria and maternal anaemia to be bi-directional. *

Do you agree or disagree with the bi-directional relationship between clinical malaria and maternal anaemia as currently presented in the model? (feel free to use other if you would like to add a comment)

Mark only one oval.

- Agree
- Disagree
- Other: ______________________

12. M11. It was recommended to add in addition of the colour coding used for the timing of health effects, symbols which can be interpreted independently of colour. The symbols are shown below the bar at the bottom of the model and were added below each outcome. We appreciate that this makes the model a lot more busy, however it would help readers when interpreting it without ability to see colour. We would like to hear what you think about this:

____

____

____

____

____

13. M12. Before moving onto child outcomes in the model, do you have any other comments regarding the maternal outcomes and their relationships?

____

____

____

____

____

Child outcomes in the model

In this section we will review the child outcomes and the relationships included in the draft model.
Any differences in lines are for visualisation purposes only. There is no different meaning between different line styles. Similarly differences in sizes of boxes also do not reflect importance, their size was purely adjusted to fit the text.

Health outcomes in the child - round 2

The main changes amongst the child outcomes are:

- the way "low birth weight" is presented and the addition of "Small for gestational age" as an outcome

-the relabeling of the short-, mid- and long-term morbidities to neonatal, infant, <5 and older child/adult morbidities.

Possible adverse effects in the child associated with *P.falciparum* exposure in utero

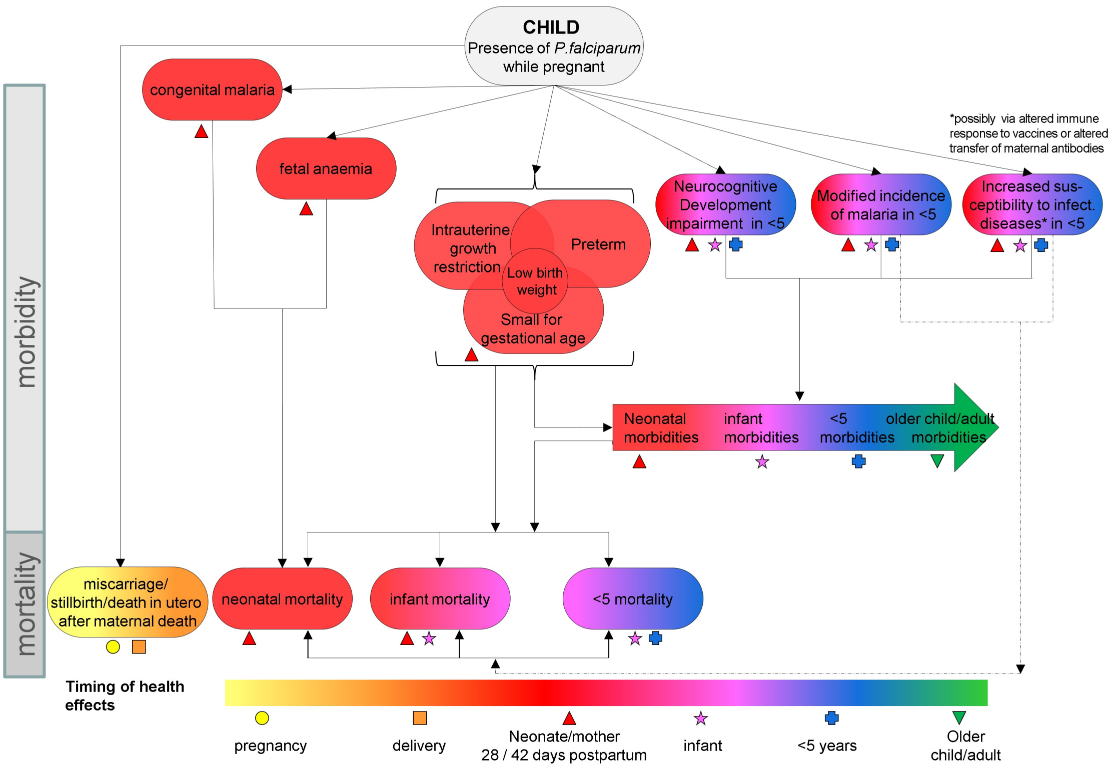

14. C1. In round 1, most experts stated that different causes of low birth weight should NOT be combined and a number of experts suggested to add in addition of preterm and intrauterine growth restriction also small for gestational age. In response we have changed the presentation of these outcomes (overlapping of preterm birth, intrauterine growth restriction and small for gestational age). If you would like to make any comments about this, please leave them here (feel free to leave empty if you are happy with the change):

15. C2. In response to comments, we added a relationship from morbidities (neonatal, infant, <5 and older child/adult) to mortalities (neonatal, infant, <5). If you would like to make any comments about this, please leave them here (feel free to leave empty if you are happy with the change):

16. C3. A number of experts have pointed out that child outcomes described at the top right - “neurocognitive development impairment in <5”, “modified incidence of malaria in < 5” and “increased susceptibility to infectious diseases in <5”- should not be separate outcomes, but within the “neonatal, infant, <5 and older child/adult morbidities”. Could you comment if you think they should rather be separate outcomes as currently in the model or included within the morbidities (with more detail given in a explanatory box)?

17. C4. Before moving on, do you have any other comments regarding the child outcomes and their relationships in the model?

Stratifiers

In the first round, we asked you rate by importance three parameters by which the model could be stratified. The response was very mixed and isn't easy to interpret. Hence we will try to rephrase our question in a different way and will also include "transmission intensity" as a additional parameter.

18. S1. Subpopulations: Please could you carefully review the following parameters by which the model could be stratified. Could you please select one or two out of the four that you consider most important. *

Tick all that apply.

- [ ] Gravidity
- [ ] HIV status
- [ ] Transmission intensity
- [ ] Timing of exposure
- [ ] Not sure
- [ ] Other: ____________________

19. Before finishing off with this second round survey of the delphi consultation, is there any other comment you would like to make?

____

____

____

____

____

Skip to section 5 (Thank you!)

Thank you!

Thank you so much for participating in this second round of our Delphi consultation. We really value your input and the time you invested in filling in this survey.

This content is neither created nor endorsed by Google.

Google Forms
