# Supplementary material for: Co-developing a comprehensive disease policy model with stakeholders: The case of malaria during pregnancy
Source: PLOS Glob Public Health. 2025 May 7;5(5):e0003775. doi: 10.1371/journal.pgph.0003775 (PMC12057981; doi:10.1371/journal.pgph.0003775)
Supplement: S4 Text — (PDF) [file pgph.0003775.s004.pdf]

Conceptual model Delphi consultation study round 1 analysis summary

Finalized: 25 November 2022

Version 2: 25 January 2023

Comments/responses to some of the findings of this analysis will be shown in italic letters.

Background summary

A total of 17 experts were approached of which 3 didn't reply, one was on maternity leave and one couldn't take part. Twelve experts from a wide range of institutions located in Africa (N=3), Asia (N=1), North America (N=3), Australia (N=2), Europe (N=2) and internationally (N=1) agreed to take part in this Delphi consultation and all experts agreed to be named as a member of the Delphi panel.

The average years of experience working in malaria in pregnancy was 16.4 years (SD=9.6) with a range between 0-34 years. The expert with 0 years of experience has a vast amount of experience in the area of health economics and of malaria in general and was approached for that reason.

75% of respondents indicated epidemiology as an area of expertise, followed by 50% clinical, 33.3% implementation research, 25% clinical trials and 8.3% mentioned either health economics, pathogenesis or disease modelling.

Section 1 Maternal outcomes

M1. Outcomes to be included in the model

1-2 as not important for inclusion
3-4 as important but not critical
5-6 as critical to include

	Clinical malaria	Severe disease	Maternal anaemia	Serious complic.	Long-term neurol. sequelae	Maternal death	Miscarriage	Stillbirth
**Answers**								
Expert 1	4	5	5	5	5	6	5	6
Expert 2	6	5	6	6	5	6	6	6
Expert 3	3	4	3	4	2	5	6	6
Expert 4	5	5	5	5	5	5	5	5
Expert 5	4	4	6	5	2	5	5	6
Expert 6	6	5	5	3	3	6	6	6
Expert 7	6	6	6	6	5	6	6	6
Expert 8	4	6	6	6	6	6	6	6
Expert 9	6	6	5	5	1	5	5	5
Expert 10	6	unsure	unsure	unsure	unsure	6	6	6
Expert 11	6	4	2	5	5	6	5	6
Expert 12	6	6	6	unsure	6	6	6	6
**Descriptive statistics**								
N answered	12	11	11	10	11	12	12	12
Average	5.2	5.1	5.0	5.0	4.1	5.7	5.6	5.8

Range (min-max)	3 - 6	4 - 6	2 - 6	3 - 6	1 - 6	5 - 6	5 - 6	5 - 6
SD	1.1	0.8	1.3	0.9	1.7	0.5	0.5	0.4
N critical to include (5,6)	8	8	9	8	7	12	12	12
% critical to include (5,6)	67%	73%	82%	80%	64%	100%	100%	100%
N import. not critic. (3,4)	4	3	1	2	1	0	0	0
% import. not critic. (3,4)	33%	27%	9%	20%	9%	0%	0%	0%
N not important (1,2)	0	0	1	0	3	0	0	0
% not important (1,2)	0%	0%	9%	0%	27%	0%	0%	0%
Keep outcome	**yes**	**yes**	**yes**	**yes**	**yes**	**yes**	**yes**	**yes**

M2. Summary of comments regarding the maternal outcomes:

- Association of many outcomes with sub-clinical/asymptomatic infections mentioned by 2 experts
  - *Response*: *asymptomatic parasitaemia has been added to the graph*
- Composite outcomes mentioned
  - *Response: composite outcomes make a lot of sense in epidemiology, however in health economics outcomes will be valued as DALYs (or QALYs) and hence require a clear description of the disease state to be able to allocate an appropriate disability or utility weight to the outcome*
- Effect modifiers, such as endemicity, gravidity or prevalent species are critical to include.
  - *Response: We are dealing with effect modifiers later in the survey and will also continue to explore this.*
- Overlap between "severe disease" (i.e. severe malaria) and "serious complications". Was pointed out by a number of experts.
  - *Response: Very helpful point, after reading up more on severe malaria, we will look at this more in the next round*
- Long term neurological sequelae, is there really any data:
  - *Response: At this point we are not yet looking into data availability*

<u>Comments asking questions/ making suggestions:</u>

- Maternal death ends day 42 post partum,
  - *Has been changed on the timeline strip*
- Miscarriage better as effect on the child:
  - *Response: It is grouped as effect on both, but was only asked once together with the maternal outcomes. This was done for no particular reason. It is definitely an outcome that affects both mother and child.*
- How would the fetal/neonatal outcome be described of a women who dies with retained pregnancy:
  - *Response: Certainly maternal death, but if stillbirth gets counted and the gestational age of the child was within the range of stillbirth, then it should contribute equally to the DALYs as stillbirth does, it was added to the model together with miscarriage, stillbirths as "fetal death after maternal death".*
- Maternal anaemia occurs unrelated to malaria and MiP can exacerbate maternal anemia. Severe maternal anemia can be associated with fetal loss directly. Then anaemia when? AT any point during pregnancy. Here it's not about the measurement but the outcome. You cannot attribute DALYs to a Hb measurement. I don't understand one statement: Anaemia is part of WHO definition of severe, so why does it have a separate box? (back to severe disease).

- ➢ *Response: agreed, Severe anaemia is indeed part of the definition of severe malaria. This has been described in more detail in the model now and also severe malaria and serious complications have been collapsed together.*

- Outcomes need a clear definition:
  - ➢ Outcomes are usually defined by clinical trial teams with some variations to be expected, we will provide an overview with description of outcomes once the model is finalized.

M3. Do you think serious complications can be combined in the model or should each of the four listed complications be shown individually?

Summary of answers

Combine	6 (50%)
Separate	2 (17%)
It depends	4 (33%)
Total	12

A number of comments in question M2 also point towards combining the maternal outcomes of serious complications. A number of experts pointed out that severe disease and serious complications shouldn't be separated. After further discussions and reading, we have combined severe disease and serious complications into one outcome. We will ask if all experts agree with this in the next round. Also thinking about how to allocated DALYs to the outcomes, it makes most sense to only include severe malaria and the possibility of long term sequelae.

M4. Would you like to suggest any other maternal outcomes to be included in the model?

Maternal outcomes that were mentioned by experts were:

- Asymptomatic parasitaemia, can be associated with adverse effects in the mother
- ➢ *Has now been added to the chart*
- Severe anaemia and placental malaria
  >severe anaemia part of definition of severe malaria, has been included there and is stated in the definition of placental malaria. Placental malaria is a relevant epidemiological and clinical outcome, however is difficult to attach a DALY to.
- Hypertensive disorders in pregnancy/ pre-eclampsia mentioned three times.
- ➢ *This outcome will be investigated further in the next round*
- Pre-conception exposure (makes mother ill, and then can lead to miscarriage as a result of preconception malaria)
- Postpartum infections due to low immunity caused by pregnancy and malaria in infection
- ➢ *We decided that this is beyond the scope of the model*
- One expert suggested to only include outcomes that have a permanent impact on QoL/ mortality not just during pregnancy and that result in substantial additional cost.
- ➢ this is a good suggestion, which will be done and justified by the economics team after the expert consultation has been completed.

M5. Before moving onto relationships, do you have any further comments?

- Might want to expand long-term neurological sequelae to simply say long term sequelae of severe disease, so not only restricted to neurological sequelae.
  - ➢ *Will be considered in the next round*
- Have specifically severe anaemia
  - ➢ *Part of definition of severe malaria*
- Exposure is a vague start to a model, Maybe say confirmed P. falciparum. Confirmation needs a definition. How do you define exposure to P.falciparum
  - ➢ *Another suggestion was presence of P. falciparum, which is a good suggestion and will be added in the next round.*
- Placental malaria
  - ➢ *Will be explored more in the next round*
- Blended colour scheme is hard to see/interpret, maybe visualize with yellow, orange, red circle, square, triangle for different periods of time.
  - ➢ *We will try and incorporate this suggestion*

M6 and M7. Combining responses on relationships maternal outcomes.

Exposure to P.falciparum → clinical malaria :

11/12 (92%) judged this relationship as correct and 1/12 (8%) as unsure. One expert asked to define exposure to P.falciparum clearly and suggested to change it to presence of the parasite.

Exposure to P.falciparum → miscarriage/stillbirth:

10/12 (83%) judged this relationship as correct and 2/12 (17%) as unsure.

Exposure to P.falciparum → maternal anemia:

11/12 (92%) judged this relationship as correct and 1/12 (8%) as unsure.

Clinical malaria →severe disease:

12/12 (100%) judged this relationship as correct. No comments.

Clinical malaria → miscarriage/stillbirth:

11/12 (92%) judged this relationship as correct and 1/12 (8%) as incorrect, other. The expert pointed out that both miscarriage/stillbirth & maternal anaemia are not only associated with clinical malaria but also asymptomatic malaria/silent infections and this should be reflected in the model.

Clinical malaria → maternal anaemia:

11/12 (92%) judged this relationship as correct and 1/12 (8%) as incorrect, other. Comment as above

Severe disease → serious complications:

9/12 (75%) judged this relationship as correct, 1/12 (8%) as incorrect, other and 2/12(17%) as unsure. One expert felt that severe disease and serious complications are synonymous. One other expert felt similarly that there is overlap between serious complications and severe disease.

Severe disease → miscarriage/stillbirth:

12/12 (100%) judged this relationship as correct. No comments.

Severe disease → maternal anaemia:

8/12 (67%) judged this relationship as correct, 2/12 (17%) as incorrect, make bi-directional, 1/12 (8%) as incorrect, change direction and 1/12(8%) as unsure. One expert said that maternal anaemia should be a form of severe disease. Other expert said the same and in addition, that maternal anaemia can make the case for severe disease, so relationship should be bi-directional

Serious complications→ long-term neurological sequelae:

10/12 (83%) judged this relationship as correct and 2/12 (17%) as unsure. No comments were made.

Serious complications→ miscarriage/stillbirths:

12/12 (100%) judged this relationship as correct. No comments.

Serious complications→ maternal death:

12/12 (100%) judged this relationship as correct. No comments.

Maternal anaemia → miscarriage/stillbirth:

9/12 (75%) judged this relationship as correct, 1/12 (8%) as incorrect, remove and 2/12(17%) as unsure. One expert felt didn't know if there were any data showing the association between anaemia and miscarriage/stillbirth.

Maternal anaemia → maternal death:

11/12 (92%) judged this relationship as correct and 1/12 (8%) as unsure. The expert noted to have anaemia more as a contributing factor to death from other factors such as post-partum haemorrhage.

- ➢ *Agreed that anaemia is more of a contributing factor to maternal death, but so are other relationships, such as clinical malaria leading to miscarriage. The way the model is currently drafted does not preclude that*

M8. If you have any comments regarding the relationships between maternal outcomes, please leave them here:

Points made:

- Exposure to P. falciparum can lead to asymptomatic malaria, which could have same outcomes as the clinical malaria
  - ➢ *This was added to the model*
- Maternal death if before delivery can cause fetal death in utero, hence add a relationship to miscarriage/stillbirth. Review the way the bracket from clinical malaria to anaemia and miscarriage/ stillbirth to improve presentation.
  - ➢ Was added to the model

M9. Would you like to suggest any other relationships between maternal outcomes to be included in the model?

- Exposure→HDoP/preeclampsia/eclampsia→maternal death/stillbirths/miscarriage (2 experts)
- Asyptomatic parasitaemia → maternal anaemia/miscarriage/stillbirths or moderate/severe anaemia combined with asymptomatic infection→ pregnancy loss
- Pre conception exposure as a risk factor, infection during pregnancy as a risk factor for malaria in the community

M10. Before moving onto child outcomes in the model, do you have any other comments regarding the relationships between maternal outcomes?

Sub-clinical infections, pre-eclampsia, Diabetes, "morbidity" and "mortality" , i think a big arrow downwards with "severity" would be helpful.

C1. Child outcomes included in the model

1-2 as not important for inclusion

3-4 as important but not critical

5-6 as critical to include

	Fetal anaemia	Congenital ma	Low birth weig	Neurocognitiv	Modified incid	Increased susc	short/mid/lon	Neonatal mort	Infant mortalit	<5 mortality
Expert 1	5	4	6	6	5	4	6	6	6	5
Expert 2	6	6	6	5	5	6	6	6	6	6
Expert 3	4	2	6	3	4	3	5	6	6	6
Expert 4	5	5	6	5	5	5	5	6	6	5
Expert 5	2	4	6	5	5	4	4	5	5	4
Expert 6	5	6	4	5	5	5	unsure	6	6	6
Expert 7	4	4	6	5	6	6	3	6	6	6
Expert 8	5	6	6	5	5	5	6	6	6	5
Expert 9	4	6	6	6	5	6	5	6	6	5
Expert 10	1	4	1	6	unsure	6	5	6	6	6
Expert 11	unsure	6	6	5	unsure	unsure	unsure	6	unsure	unsure
Expert 12	3	3	6	5	5	5	5	6	6	6
N answered	11	12	12	12	10	11	10	12	11	11
Average	4.0	4.7	5.4	5.1	5.0	5.0	5.0	5.9	5.9	5.5
Range (min-max)	1 - 6	2 - 6	1 - 6	3 - 6	4 - 6	3 - 6	3 - 6	5 - 6	5 - 6	4 - 6
SD	1.4	1.3	1.4	0.8	0.4	1.0	0.9	0.3	0.3	0.7
N critical to include (5 or 6)	5	6	10	11	9	8	8	12	11	10
% critical to include (5 or 6)	45%	50%	83%	92%	90%	73%	80%	100%	100%	91%
N important not critical (3 or 4	4	5	1	1	1	3	2	0	0	1
% critical to include (3 or 4)	36%	42%	8%	8%	10%	27%	20%	0%	0%	9%
N not important (1 or 2)	2	1	1	0	0	0	0	0	0	0
% not important (1 or 2)	18%	8%	8%	0%	0%	0%	0%	0%	0%	0%
Keep outcome	yes	yes	yes	yes	yes	yes	yes	yes	yes	yes
check	11	12	12	12	10	11	10	12	11	11

C2. Summary of comments regarding child outcomes:

By outcome:

Fetal anaemia: One expert rated it as not important and commented that the data is generally not available. Another expert wondered what its impact is and that it might differ between Pf and Pv.

Congenital malaria: one expert noted that it's a very rare outcome and molecular detection studies often overestimate the prevalence of congenital malaria

Low birth weight: 3 experts suggest that it should be split into SGA, IUGR and preterm birth as the main cost of LBW is due to prematurity.

short/mid/long-term morbidities: One expert is unsure how it could be defined. Another said that it could be hard to map by cause of LBW, but really important (hopefully more data in the future).

For all other outcomes there were no specific comments and all information is summarized in the table above.

Other points made:

Consider effect modifiers such as transmission, species and gravidity→ *we looked at effect modifiers later in the questionnaire*

Two experts pointed out to have arrows from morbidities to increased mortality

What is the modified incidence of malaria was a question asked→ *there is evidence that the incidence of malaria during pregnancy is modified in <5 years if exposed to malaria during pregnancy compared to those who were not. See https://pubmed.ncbi.nlm.nih.gov/31481075/*

C3. Summary if "low birth weight" should be separated

Combine	0
Separate	12
It depends	0
changes suggested	2

Main comments were to add as much detail as possible, as mortality, morbidity as well as costs differ between the different groups. Experts also suggested to add small for gestational age (SGA). A change in presentation was recommended by one expert.

C4. Any other child outcomes suggested to be included in the model:

- Three experts mentioned SGA, and also don't miss PTD that is not LBW, also newborn can neither be lbw nor preterm but be SGA, for example use of intergrowth-21
- infant anaemia not only fetal anaemia
- Childhood stunting/ wasting was mentioned by 2 as result of risk of malaria and other childhood illnesses→ *covered in morbidities*
- Not only modified incidence of 5 malaria, but also of <5 severe malaria
- Metabolic diseases in later life→*covered in long term morbidities*
- Modified vaccine efficacy in infant after MiP in utero → *covered in comment to susceptibility of infectious disease*

Other comments were:

- Skilled attendance at birth.

- G6BD deficiency (*is that an outcome or an risk factor?).*

C5. Before moving onto relationships between child outcomes in the model, do you have any other comments regarding the child outcomes?

- Child outcomes might be different for female and male children
- Exclusion of twin babies when assessing LBW
- It was suggested to replace the short, medium and long term morbidities with neonatal, U5, older childhood and adulthood morbidities in boxes and remove explanatory text
- Have both maternal and child outcomes on one page.

C6 and C7. Combining comments from C7 with responses in C6 by relationship

Exposure to P. falciparum --> fetal anaemia:

9/12 correct (75%), 3 unsure, 1 expert would like to have exposure clearly defined.

Exposure to P.falciparum --> congenital malaria

10/12 correct (83%), 2 unsure, no specific comments

Exposure to P.falciparum --> low birth weight

10/12 correct (83%), 2 unsure, no specific comments, but to remove LBW as it is and stratified.

Exposure to P.falciparum --> neurocognitive development impairment in <5

9/12 correct (75%), 2 unsure, 1 incorrect. Neuro impairments are a morbidity, so they don't really lead to morbidity, the expert who judged it as incorrect said that evidence is unclear about the strength and magnitude of the association. It's ok to include, however uncertainty might be large.

Exposure to P.falciparum --> modified incidence of malaria in <5

10/12 correct (83%), 2 unsure, 1 expert would like to have exposure and modified incidence of malaria clearly defined.

Exposure to P.falciparum --> increased susceptibility to infectious diseases in <5

8/12 correct (67%), 3 unsure, 1 incorrect, one expert would like to have exposure clearly defined. The expert who judged it as incorrect said that evidence is unclear about the strength and magnitude of the association. It's ok to include, however uncertainty might be large

Fetal anaemia --> neonatal mortality

8/12 correct (67%), 3 unsure, 1 incorrect. The expert who judged it as incorrect said that evidence is unclear about the strength and magnitude of the association. It's ok to include, however uncertainty might be large

Congenital malaria --> neonatal mortality

10/12 (83%) correct, 1 unsure, 1 incorrect. The expert who judged it as incorrect said that evidence is unclear about the strength and magnitude of the association. It's ok to include, however uncertainty might be large

Low birth weight --> neonatal mortality

11/12 (92%) correct, 1 incorrect (remove). The expert who said remove, says LBW should be stratified.

Low birth weight --> infant mortality

11/12 (92%) correct, 1 incorrect (remove). The expert who said remove, says LBW should be stratified.

Low birth weight --> <5 mortality

9/12 (75%) correct, 2 unsure, 1 incorrect (remove). The expert who said remove, says LBW should be stratified.

Low birth weight --> short/mid/long-term morbidities

10/12 (83%) correct, 1 unsure, 1 incorrect (remove). The expert who said remove, says LBW should be stratified.

Neurocognitive development impairment in <5 --> short/mid/long-term morbidities

9/12 (75%) correct, 2 incorrect, specify, 1 incorrect remove. 1 expert who said incorrect other says that arrows should reflect that preterm is associated with neurocogn dev impairment, other expert says that neurocognitive impairment is a morbidity and doesn't lead to one.

Modified incidence of malaria in <5 --> short/mid/long-term morbidities

8/12 (67%) correct, 3 unsure, 1 incorrect (other). The expert who judged it as incorrect said that evidence is unclear about the strength and magnitude of the association. One expert was unsure about what modified incidence of malaria is.

Increased susceptibility to infectious diseases in <5 --> short/mid/long-term morbidities

10/12 (83%) correct, 1 unsure, 1 incorrect (other). The expert who judged it as incorrect, other said that evidence is unclear about the strength and magnitude of the association. The expert who selected unsure says that for many associations it is biologically plausible but unsure about the evidence supporting it.

Modified incidence of malaria in <5 --> neonatal mortality

8/12 (67%) correct, 2 unsure, 1 incorrect remove, 1 incorrect other. The expert who judged it as incorrect, other said that evidence is unclear about the strength and magnitude of the association. Otherwise no comments

Modified incidence of malaria in <5 --> infant mortality

8/12 (67%) correct, 2 unsure, 1 incorrect remove, 1 incorrect other. The expert who judged it as incorrect, other said that evidence is unclear about the strength and magnitude of the association. Otherwise no comments

Modified incidence of malaria in <5 --> <5 mortality

8/12 (67%) correct, 3 unsure, 1 incorrect, other. The expert who judged it as incorrect, other said that evidence is unclear about the strength and magnitude of the association. Otherwise no comments. One expert was unsure what modified incidence of malaria in <5 was.

Increased susceptibility to infectious diseases in <5 --> neonatal mortality

8/12 (67%) correct, 1 unsure, 2 incorrect, remove, 1 incorrect, other. The expert who judged it as incorrect, other said that evidence is unclear about the strength and magnitude of the association. Otherwise no comments. The two who said remove, only said it specifically for neonatal mortality, they judged it for infant and <5 mortality 1 as unsure and one as correct.

Increased susceptibility to infectious diseases in <5 --> infant mortality

9/12 (75%) correct, 2 unsure, 1 incorrect, other. The expert who judged it as incorrect, other said that evidence is unclear about the strength and magnitude of the association. Otherwise no comments.

Increased susceptibility to infectious diseases in <5 --> <5 mortality

9/12 (75%) correct, 2 unsure, 1 incorrect, other. The expert who judged it as incorrect, other said that evidence is unclear about the strength and magnitude of the association. Otherwise no comments.

C8. If you have any comments regarding the relationships between child outcomes, please leave them here:

Comment made were the following:

Fetal anaemia can lead to outcomes other than neonatal mortality (e.g. neurocognitive development impairment or infant mortality).

Does increased susceptibility to U5 diseases operate by a pathway independent of preterm birth and LBW? What is "modified incidence?" Higher or lower? Could this be asked in the survey?

C9. Would you like to suggest any other relationships between child outcomes to be included in the model?

Severe malaria

SGA, non preterm, non LBW SGA.

C10. Before moving onto stratifiers in the model, do you have any other comments regarding the relationships between child outcomes?

No further comments

S1. Subpopulations:

	HIV status	Gravidity	Timing exposure
Most important	4 (33%)	8 (67%)*	2 (17%)
Average important	4 (33%)	1 (8%)	6 (50%)
Least important	4 (33%)	2 (17%)	3 (25%)
Unsure	0	1 (8%)	1 (8%)

Gravidity, HIV status and timing of exposure

S2. Are there any additional parameters which you think are important and by which the analysis should be stratified? If possible, please support your suggestion with evidence.

- Transmission intensity mentioned 7 times
- General nutritional status of mother/ food security 3 times
- Infant sex mentioned 2 times
- Drug resistance mentioned once→ difficult to deal with, but data used in model should definitely reflect this appropriately
- Sickle cell disease mentioned once → would likely be reflected in the data used in the model

- Socioeconomic status mentioned once→ should be reflected in the data on both cost and outcomes side of the model
- PV, Pf or both areas mentioned once→ good point, model will focus on Pf for now
- Artemisin or quinine based treatment mentioned once → model will deal with that.
