# Supplementary material for: Co-developing a comprehensive disease policy model with stakeholders: The case of malaria during pregnancy
Source: PLOS Glob Public Health. 2025 May 7;5(5):e0003775. doi: 10.1371/journal.pgph.0003775 (PMC12057981; doi:10.1371/journal.pgph.0003775)
Supplement: S5 Text — (PDF) [file pgph.0003775.s005.pdf]

Conceptual model Delphi consultation study round 2 analysis

Finalized: 19 July 2023

Comments/responses to some of the findings of this analysis will be shown in italic letters.

Action points for me will be shown in green and decision points to still be decided by the expert panel in yellow.

Section 1 Maternal outcomes

M1. One expert recommended to use instead of “Exposure to P.falciparum while pregnant” the term “Presence of P.falciparum while pregnant”. We felt that this is a good suggestion and have adapted this term in the model. If you would like to make any comments about this or would like to suggest something different, please do this here (feel free to leave empty if you are happy with the change)

Summary with comments and potential action points (in green):

10/12 (83%) experts were happy with the wording. The following comments were made:

- the foetus may be directly "exposed" to malaria antigens in utero (if they cross the placenta) as opposed to the "presence of Pf per se"
    - *the wording can be changed to “Presence of/ exposure to P. falciparum in utero” for the foetus*
- 'Exposure' is a broad term, as it may not necessarily result in a blood-stage infection. Consider the "presence of blood-stage plasmodium parasites", which reflects the stage that causes harm and reflects all plasmodium species infecting humans. Furthermore, it broadens it beyond falciparum malaria (if that is part of your intention, otherwise it could be left as Pf).
    - *This is a really good suggestion, however for simplicity, we prefer to keep it as it currently is. We will, however, aim to mention this in the accompanying paper.*

M2. Many experts suggested to add asymptomatic parasitaemia (or silent infection, or sub-clinical infection etc.) as a separate outcome box. We have added it as a separate outcome box. Relationships from asymptomatic parasitaemia lead to clinical malaria, maternal anaemia, hypertension disorders of pregnancy and miscarriage/stillbirths. If you would like to make any comments regarding this new outcome and its relationships, please leave them here (feel free to leave empty if you are happy with the change):

Summary with comments and potential action points (in green):

All experts (12/12, 100%) agree with this suggestion. One expert commented to:

- use asymptomatic parasitaemia and if possible in brackets silent infection for the more general population.

- *As this model is less targeted at the general population, we will keep the term asymptomatic parasitaemia.*

M3. Many experts suggested to add placental malaria as a separate box. This has now been included with clinical malaria and asymptomatic parasitaemia as both can occur with and without placental malaria. The purpose of this model is to use it as a cost effectiveness model for malaria in pregnancy, hence outcomes will in the end be valued as DALYs and for both placental malaria and asymptomatic parasitaemia no DALY weights exist. We however felt that they show an important pathway, essential to depict in this model, even if they do not count towards the final DALY outcome. To not overcomplicate the model, we have included placental malaria not separately, but together with both asymptomatic parasitaemia and clinical malaria. Hence these two outcome boxes now read "asymptomatic parasitaemia with & without placental malaria" and "clinical malaria with & without placental malaria". If you would like to make any comments about this, please leave them here (feel free to leave empty if you are happy with the change):

Summary with comments and potential action points (in green):

9/12 (75%) experts agree with our suggestion. The three experts who didn't fully agree, made the following comments:

- I am more in favor of having a separate box for placental malaria, as I have the sense to considerer it a different pathway from the other.
- If the placental malaria will not be systematically assessed at delivery and the data considered, I would suggest that we completely remove it from the model and leave only symptomatic and clinical malaria. In fact the way it is currently presented, it does not have any added value to the model as it seems to be neutral.
    - *This is probably correct, but for completeness of including viewpoints of all experts that will look at this model, we will keep it as it currently is. We will however elaborate this point in more detail in the accompanying paper.*
- There is work to show that early diagnosis and treatment (which has economic costs) can result in a negative placenta at delivery. Placental malaria alone cannot tell you if a woman had P. falciparum in pregnancy. Studies don't always check for both.
    - *we completely agree with this, which is another reason we felt we shouldn't have placental malaria as a separate box and included it with asymptomatic and clinical malaria as otherwise we would have to have boxes for the different possible combinations (i.e. clinical malaria without placental malaria, clinical malaria with placental malaria, asymptomatic parasitaemia without placental malaria, asymptomatic parasitaemia with placental malaria).*

M4. In the previous model maternal outcomes included "severe disease", "serious complications" and "long-term neurological sequelae". It was suggested by several experts that the serious complications are already implied in the definitions of severe malaria and are hence not required as a separate outcome. We have now relabelled "severe disease" as "severe malaria" and removed the box "serious complications"

and included a box with the WHO definitions of severe malaria, which also includes severe anaemia. If you would like to make any comments about this, please leave them here (feel free to leave empty if you are happy with the change):

Summary with comments and potential action points (in green):

10/12 (83%) experts agreed with this suggestion. The following comments were made:

- One expert pointed out that not all signs of severe malaria are equally associated with mortality.
  - ➢ *This is very true and while at this point in time it is unlikely that we will have the level of detail of data to be able to differentiate the severe disease outcomes in the model. However, we will highlight in the discussion of the paper that hopefully with time it will be possible and highlight the importance of having more granular data. The paper of the conceptual model will at first focus on a "would like to have" model if science and data caught up and then we will have to look at a model that is more realistic to populate with currently available data.*
- One expert pointed out that explanation boxes such as the one describing the definition of severe malaria should go in the footnote.
  - ➢ *We have moved explanations to bottom of the model.*

M5. It was recommended to use "long term sequelae" instead of "long term neurological sequelae" as other organs can also suffer from long term sequelae for example chronic kidney sequelae. However, other experts pointed out that consequences of cerebral malaria are quite different from other long term disabilities caused by severe malaria. We would like to ask you which of the following you think is most suitable to describe long term sequelae of severe malaria:

Summary with comments and potential action points (in green):

Descriptive summary table:

To use long term neurological sequelae only	1
To use long term sequelae only	6
To have both as separate outcome boxes	4
Don't know/ unsure	1
Total	12

The result is not completely clear, but 6/12 (50%) experts said they would use long term sequelae only. The following comments were made:

- What about "long term neurological and other sequelae" ?
  - ➢ *Good suggestion. We will use this suggestion in the model, which will save us the explanation box.*
- I assume this will affect your DALY calculation. If you can assign one DALY for "long term sequalae" (regardless of whether it is neurological, kidney function, etc), then just one box

would be fine. Otherwise, I guess you need to have some separate boxes for each major consequences.

- ➢ *That's very true. It will require some careful review of the burden of disease study disability weight estimates and match long term sequelae such as chronic kidney disease to descriptions in the burden of disease study health states. A number of outcomes currently in the same boxes might need to be distinguished over time, data availability permitting.*

M6. One expert commented that maternal death if before delivery can cause fetal death in utero, hence we have added a relationship from maternal death to miscarriage/stillbirth and have extended that box to include also "death in utero after maternal death". If you would like to make any comments about this, please leave them here (feel free to leave empty if you are happy with the change):

Summary with comments and potential action points (in green):

10/12 (83%) experts agree with this suggestion. The following comments were made:

- One expert noted that it overcomplicates a relatively rare event, especially in the light of the total burden of miscarriage and stillbirth, but doesn't disagree with the suggestion as such.
  - ➢ *We completely agree with this being a relatively rare event and it having an extremely limited contribution to overall DALYs. Including it serves more the completeness of the model*
- One expert also suggested to say "Death in utero with maternal death" instead of "Death in utero after maternal death"
  - ➢ *we will simplify to "Death in utero" to avoid confusion as in a later comment (to question C4) it appeared unclear to an expert that the comment "with maternal death" is meant to only relate to death in utero.*

M7. A number of experts suggested that "hypertension disorders of pregnancy" including pre-eclampsia and eclampsia should be added to the model. Could you please indicate if hypertension disorders of pregnancy should be included in the model by rating its importance for inclusion on a scale 1-6.
We consider:
1-2 as not important for inclusion
3-4 as important but not critical
5-6 as critical to include

You will have an opportunity to comment further if needed in question M9.

**Descriptive statistics**	
N answered	11
Average	3.5
Range (min-max)	2 - 6
SD	1.2
N critical to include (5 or 6)	2
% critical to include (5 or 6)	18%

N important not critical (3 or 4)	7
% critical to include (3 or 4)	64%
N not important (1 or 2)	2
% not important (1 or 2)	18%
Include outcome	**TBD**

- ➢ *Including hypertension disorders of pregnancy does not seem to have a huge support by the experts with only 18% judging it as critical to include and 64% as important but not critical and 18% as not important. It has neither achieved our criteria for inclusion nor exclusion. I am also aware that there seems to be a difference in opinion between experts working in low endemicity settings, where evidence for this outcomes mostly originates from.*
- ➢ *We aim to determine this during our final discussion and debrief we will be holding over zoom.*

M8. For the relationships (indicated by the arrows) between "hypertension disorders of pregnancy" and other outcomes shown above (or on page X of the model draft), please indicate whether you think they are correct or incorrect (indicating what correction is needed)

See summary below in M9.

M9. If you have any comments regarding the added outcome "hypertension disorders of pregnancy" and its relationships to other outcomes, please leave them here:

Combining comments from M9 with responses in M7 and M8

Summary with comments and potential action points (in green):

Asymptomatic parasitaemia -> hypertension disorders of pregnancy:

9/12 (92%) judged this relationship as correct and 3/12 (8%) as unsure.

Clinical malaria → hypertension disorders of pregnancy:

9/12 (92%) judged this relationship as correct and 3/12 (8%) as unsure.

Hypertension disorders of pregnancy → miscarriage/ stillbirth:

12/12 (100%) judged this relationship as correct

Hypertension disorders of pregnancy → maternal death:

12/12 (100%) judged this relationship as correct

- ➢ *while experts are unsure if to include hypertensive disorders of pregnancy in the model, the relationships were all judged as correct by the panel.*

- One experts noted limited data suggests that hypertensive disorders is less frequent in high vs low endemicity areas
- One expert says it is a very rare outcome of malaria infection and doesn't think that it operates after delivery

- *There is a chance of hypertension disorder, pre-eclampsia and eclampsia to start in the 4 weeks after delivery. It's not well known by the medical profession and understudied to be able to judge prevalence:* https://pubmed.ncbi.nlm.nih.gov/32761267/
- *To reflect this not only with the symbol but also with the label of the box, we will change the label to "hypertension disorder of pregnancy and post-partum"*

- One experts isn't convinced of the evidence base for this association
- One experts says although associated with MiP, if it makes model too complicated hypertensive disorder during pregnancy can be omitted.
- One expert suggests the need to distinguish hypertensive disorders of pregnancy further into pre-eclampsia/ eclampsia and gestational hypertension
  - *We understand the importance of distinguishing between pre-eclampsia/eclampsia and gestational hypertension in terms of both costs and DALYs, especially for low endemicity settings. To avoid further complicating the model it will be clarified in a footnote and in more detail in the discussion of the paper.*
- One expert pointed out that there are also other causes of hypertension
- One expert points out that the effect may be modified by gravidity: https://www.ncbi.nlm.nih.gov/pmc/articles/PMC8077872/
  - *Comparing answers and comments from different experts and their focus areas of research, there appeared to be a less strong conviction of the importance of hypertension disorder of pregnancy for researchers focusing on high endemicity settings only, compared to those working in low endemicity settings. Hence I am reluctant to remove the outcome from the model, as the model should also be representative for low endemicity settings and evidence points towards that it is more relevant for low endemicity settings. There will be an opportunity to discuss inclusion and exclusion of hypertension disorders during pregnancy during the final debrief and discussion held over zoom.*

M10. A number of experts suggested the relationship between clinical malaria and maternal anaemia to be bi-directional.

Do you agree or disagree with the bi-directional relationship between clinical malaria and maternal anaemia as currently presented in the model? (feel free to use other if you would like to add a comment)

33% (N=4) of experts agree with the suggested bi-directional relationship and 67% (N=8) disagree with it.

- *The comments suggest that this should be clarified: we did not mean to say that maternal anaemia causes clinical malaria, but contributes to the progression from asymptomatic malaria to clinical malaria. An arrow to indicate that anaemia contributes to the progression from asymptomatic malaria to clinical malaria is suggested. There will be an opportunity to discuss this during the final debrief and discussion held over zoom.*

M11. It was recommended to add in addition of the colour coding used for the timing of health effects, symbols which can be interpreted independently of colour. The symbols are shown below the bar at the bottom of the model and were added below each outcome. We appreciate that this makes the model a lot more busy, however it

would help readers when interpreting it without ability to see colour. We would like to hear what you think about this:

10/12 (83%) experts agree with the presentation. 1/12 has no preference over having the symbols or not and 1/12 found it the symbols too distracting

➢ *we will keep the symbols as suggested in the model.*

M12. Before moving onto child outcomes in the model, do you have any other comments regarding the maternal outcomes and their relationships?

<u>Summary with comments and potential action points (in green):</u>

- The lines should be normal lines rather than different line types and having little half circles to show where one line crosses past another line.
  ➢ *We agree with this comment and will make sure that all lines are the same*
- Gravidity should be considered in each relationship of the model as the effect is modified by it.
  ➢ *Thank you for this comment. That's absolutely correct.*

C1. In round 1, most experts stated that different causes of low birth weight should NOT be combined and a number of experts suggested to add in addition of preterm and intrauterine growth restriction also small for gestational age. In response we have changed the presentation of these outcomes (overlapping of preterm birth, intrauterine growth restriction and small for gestational age). If you would like to make any comments about this, please leave them here (feel free to leave empty if you are happy with the change):

10/12 (83%) experts agree with the presentation.

➢ *We will keep the presentation as it is*

<u>Summary with comments and potential action points (in green):</u>

- One expert suggest to replace Intra uterine growth restriction with small for gestational age and one expert suggests that SGA could be removed, as most likely it will only be from a BW measurement at birth and hence a marker of increased risk of IUGR.
- I'd be interested to see how the team defines IUGR. BW<10th centile (i.e., SGA). Change in growth velocity? Both? Realistically, what will be most available is SGA based on BW centiles (i.e., a single measurement of weight or other, at birth). In which case SGA is simple a marker of increased risk of IUGR, rather than a separate entity. In which case you do no need to have SGA in the chart, but perhaps explain in a footnote how IUGR could be defined and mention SGA there.
  ➢ *definition of outcomes is often problematic for economic models, as we often have to go with what the trial teams have decided, which frequently differs between teams and studies. We can only influence if involved in the planning stage of a study, which is rarely the case. Hence, when pooling results of multiple studies for a cost-effectiveness analysis of all available evidence, we usually show in an appendix table the different definitions of outcomes by study. (if there is a difference).*

C2. In response to comments, we added a relationship from morbidities (neonatal, infant, <5 and older child/adult) to mortalities (neonatal, infant, <5). If you would like to make any comments about this, please leave them here (feel free to leave empty if you are happy with the change):

Most experts agree with our suggestion (8/12, 67%).

Summary with comments and potential action points (in green):

- Visually, it is odd that i) the morbidities are all lumped together in one symbol, while the mortalities are individual and ii) the morbidities are in a big arrow, which leads to nothing, while all the other arrows indicate a causal relationship. This is just confusing from a graphic design perspective because it is inconsistent internally
  - ➢ *i) We will think about a way of presenting this more coherently by combining the mortalities into one box as well. The point of the arrow used for the other morbidities is to reflect the potential progressive nature of these morbidities over a lifetime ii) In reality these morbidities can range quite widely and we will not be able to define them for this model. But we would like to pull attention to them and that for example being born prematurely can lead to a wide ranging spectrum of morbidities during all stages of life.*
  - ➢ *We will discuss the presentation of the morbidities during the final debrief and discussion held over zoom.*
- Shouldn't the line be coming down from the middle of the morbidity arrow rather than seeming to come from the back of the arrow (at the neonatal end)?
  - ➢ *Fair point. We will adjust that.*
- as long as you can deal with the overlap in age (e.g. <5 includes neonates and infants)
  - ➢ *The main point of presenting the morbidities in the model is to pull attention to them. It will be difficult to define them for any given model.*

In addition the following two questions were asked:

- q1.Most malaria deaths are in children under 5y and this malaria is detectable. Instead of P. falciparum detection in infant/child a proxy "modified malaria incidence" is being used. This model will distinguish maternal malaria in pregnancy and/or "modified malaria incidence" as related to infant/child death, without P.falciparum presence in infant/child being confirmed?
  - ➢ *we have looked at a number of studies that suggested the relationship between placental malaria or parasitaemia during pregnancy with infant malaria as being non-linear, which is the reason we opted to say "modified malaria incidence in <5". We have now searched the literature for systematic reviews on this topic and found two (Kakuru et al 2019 and Park et al 2020). Kakuru et al concluded that there is some evidence of increased risk of malaria in infancy if infant was exposed to placental malaria or parasitaemia during pregnancy, but still more work is needed to either confirm or exclude this association between MiP and malaria in infancy. Park et al looked at the association of MiP and malaria in children and concluded that there is an increased risk malaria in young children if exposed to placental malaria or parasitaemia during pregnancy. We are hence suggesting to change the label of this field in our model from "modified incidence of malaria in <5" to "increased incidence of malaria in <5", but we will consult the experts during the meeting about any evidence suggesting that the previous title is more appropriate.*

- q2. Does infant/child morbidity exclude P.falciparum? What about blood transfusion for severe malaria?
    - *We are highlighting the main morbidities with clearer evidence in the boxes and the large arrow box reflects the other morbidities. We will change the name in the large arrow to "other morbidities" to make this clearer. A blood transfusion would be a consequence in a % of patients with severe malaria / anaemia, to which a cost will be attached. Adverse consequences of blood transfusion will probably go beyond the scope of this model, given the rarity of the blood transfusion in the first place combined with the rarity of events following blood transfusion. But it should certainly be mentioned in the accompanying paper.*

C3. A number of experts have pointed out that child outcomes described at the top right - "neurocognitive development impairment in <5", "modified incidence of malaria in < 5" and "increased susceptibility to infectious diseases in <5"- should not be separate outcomes, but within the "neonatal, infant, <5 and older child/adult morbidities". Could you comment if you think they should rather be separate outcomes as currently in the model or included within the morbidities (with more detail given in an explanatory box)?

Combine	3
Separate	7
Other	1
No comment	1

The majority of experts (7/10, 70%) agree with the current presentation in the model shown and don't think it should be combined.

<u>Summary with comments and potential action points (in green):</u>

- I think any detail belongs in notes, not another box on this figure. I think you could make the fat morbidities arrow much bigger and fatter, simplify it, and integrate the three specific morbidities into it. For example, why not just write at the top of the arrow "Morbidities, including:" and then bulleted list of the three specific ones? The word "morbidities" does not need to be repeated 4 times in the fat arrow, and the words "in <5" may not need to be repeated 3 times. The words "neonatal", "infant", "<5", and "older children" could perhaps just run along the bottom of the arrow. I may be wrong, but I think this would actually result in a simpler figure (with one much bigger big arrow)
    - *including the three specific morbidities into the large arrow was overall rejected by the experts, however the suggestion to simplify the presentation of the large arrow, by not repeating the word 'morbidities' makes a lot of sense and we will include that in our model.*

C4. Before moving on, do you have any other comments regarding the child outcomes and their relationships in the model?

Summary with comments and potential action points (in green):

- Its odd that there isn't a clearer outcome of stillbirth/miscarriage/pregnancy loss, unless that was meant to be captured on the maternal form (though I don't see it). I mean specifically independent of maternal death, i.e. mom lives but miscarries owing to antenatal malaria. There is a pretty clear causal relationship between antenatal parasitization (see https://pubmed.ncbi.nlm.nih.gov/28967610/) and pregnancy loss that this should surely appear somewhere independent of maternal death?
  - ➢ *thanks for this comment, it's helpful to know that this wasn't clear with the box. The box is referring to stillbirth and miscarriage in the first place and we added after a comment the rare event of death in utero after maternal death. The after maternal death only refers to death in utero. We have removed "after maternal death" in the box and it now only reads death in utero. The arrows leading to the box should make the it clear.*
- As some commented, physical development impairment can be caused. And fetal anaemia can be the cause of physical and neurocognitive developlement impairment (i.e., an arrow from fetal anaemia to development impairement can be drawn).]
  - ➢ *discuss possibility of adding an arrow from fetal anaemia to physical and neurocognitive development impairment during meeting. And also the possibility of having an arrow to other morbidities from fetal anaemia as well as from congenital malaria.*
- I think it's really confusing to have arrows that flow upwards. If the two separate boxes for modified incidence of malaria and increased susceptibility to diseases remain, I think their arrow flowing to mortality needs to be modified so that it flows into the top of the mortality boxes, not the bottom. I think the lines also need to be of the same style (e.g. solid) if you are not trying to indicate a different strength of relationship. Also, the arrow that flows left out of the main "Child" box comes from a different part of the box from all the other arrows, which sort of gives the impression that it means something different.
  - ➢ *Bring arrow coming from bottom to mortality to the side of the now combined mortality box*
  - ➢ *We will ensure the lines of the arrows are the same (solid) in the next model to avoid confusion about the strength of relationship indicated by different lines.*
  - ➢ *Add additional arrow in top left corner from child box to miscarriage*

S1. Subpopulations: Please could you carefully review the following parameters by which the model could be stratified. Could you please select one or two out of the four that you consider most important.

Summary with comments and potential action points (in green):

Gravidity	10
Transmission intensity	8
Timing of exposure	3
HIV status	2

11 out of 12 experts voted for 2 parameters and 1 expert for only 1.

- ➢ *Gravidity and transmission intensity are considered the most important parameters by which the model should be stratified. Of course, both HIV and timing of exposure will still*

be highly relevant and depending on context and intervention should be considered for inclusion into the model and all parameters will be discussed in the accompanying paper.

Before finishing off with this second round survey of the delphi consultation, is there any other comment you would like to make?

Summary with comments and potential action points (in green):

- In S1 above, Timing to exposure is also important as well the type of Transmission (eg seasonal versus perineal)
- I think there are some cosmetic things you could do to make the model visually come across as smaller and simpler. I suspect you could even get both the maternal and child versions on a single sheet just by moving the boxes and arrows around a little (and especially if you remove the extra child morbidity boxes).
    - ➢ *see if both maternal and child outcomes can go onto one page.*
- S1: Gravidity may be better than transmission intensity, as you will have the data by gravidity in manuscripts. It will be much harder to get detailed data by transmission intensity per study as this is less frequently reported. If you have to be exposed to malaria to be protected in the next pregnancy, then gravidity effects are a proxy for transmission. Timing of exposure is increasingly recognised as very important, but not sure you will find a lot of good-quality studies that separate data by trimester. HIV is clearly important, but it needs more data, like are they on ARVs?, when were ARVs were initiated (before or during pregnancy), CD4 counts etc. So all four are very important, but quality data may only exist for gravidity (and hopefully timing [trimester] of exposure).
    - ➢ *responses to this and the previous question clearly show that all of these factors are really important. Hence the analysis will need to ensure when using this model that it is populated with appropriate data depending on intervention, context, and as well data availability by each of these factors. If possible the model should be populated with data stratified by gravidity and by transmission context. Trials in the future should be encouraged to collect high quality data on outcomes that separate by trimester of exposure as well as better data in HIV positive women providing more context such as CD4 count and ARV use.*
