# Supplementary material for: Co-developing a comprehensive disease policy model with stakeholders: The case of malaria during pregnancy
Source: PLOS Glob Public Health. 2025 May 7;5(5):e0003775. doi: 10.1371/journal.pgph.0003775 (PMC12057981; doi:10.1371/journal.pgph.0003775)
Supplement: S6 Text — (PDF) [file pgph.0003775.s006.pdf]

# Consensus meeting Delphi Panel summary

## Summary both meetings:

Attendees meeting 1: 4 experts plus 2 steering committee attendee

Attendees meeting 2: 5 experts plus 1 steering committee attendee

9/12 experts attended (75%)

**Other suggestions:**

- Change triangle from presence of P.falciparum while pregnant to presence of P. falciparum during pregnancy✓
- Add green to mortality box✓

**Question 1: Hypertension disorders of pregnancy**

- Keep in: 9/9
- split into hypertension, pre-eclampsia and eclampsia: split 4/9, don't split 5/9
- might be best to just have eclampsia and pre-eclampsia which are biologically much more relevant than gestational hypertension, but data might be difficult to find, as often lumped together with much more prevalent outcome of gestational hypertension
- reason for not splitting: difficult to get the right diagnosis. For example distinguish between severe malaria and eclampsia might be difficult.
- Hypotension is linked to severe malaria (a manifestation of it)
- There is more hypertension disorder in pregnancy in Africa than was thought , while hypertension is always measured but often doesn't end up in the dataset.
- should be there because of consequences, Case control study (4) systematic review in Africa of hypertensive disorder and MiP 2.67 OR higher hypertensive disorder in women with MiP.
- Likely that Africa has much higher rates of hypertension than Asia (also see African Americans for example).
- Outcomes such as death and stillbirth/miscarriage same, but there are other outcomes from pre-eclampsia/eclampsia that are not captured, like mental health, stroke, other organ damage like liver, kidney, brain; also later in life heart disease, high blood pressure, kidney disease, stroke

➔ keep hypertension disorder of pregnancy, add a box to long term complications of hypertension disorders of pregnancy ✓

➔ Change colours to slightly softer to make font better legible✓

**Question 2: Is there a causal link from maternal anaemia to clinical malaria**

- Yes=keep arrow: 1/9, no remove arrow: 8/9 ✓
- high risk of confounding for progression.
- Difference between association and causal pathway

**Question 3: Morbidities**

- Oval shape: yes 7/9, no 2/9 ✓
- arrow from grey child box to other morbidities: yes 8/9, no 1/9 ✓

- arrow from congenital malaria to other morbidities: yes 9/9✓
- arrow from fetal anaemia to other morbidities: yes 9/9✓
- Paper should really highlight and discuss the potentially progressive nature of these morbidities
- Ensure that it is clear that these other morbidities are not going to be quantified in an cost effectiveness paper, for the paper on the model it is really nice to have though.
- ensure it is clear that oval box of other morbidities is sequelae of the outcome that lead into it, but also potential any other morbidities that haven't been thought off yet or haven't been studied sufficiently yet
- have some recognition in text about the nature of lifelong consequences of morbidities
- less nephrons in fetus if mum exposed to MiP, huge, chronic kidney disease

**Question 4: Modified incidence of malaria in <5 to increased incidence**

- yes 9/9 ✓

**Other comments**

- Important outcome of this work is to identify areas that are low in data and share with research community.
- Gravidity, Low and high endemicity are important factors to take account of for each of the outcomes in the model.
- Probably not feasible at this stage but with foresight into the future to move away from a one size fits all model
- association between sickle cell treat, seem to modify the risk in a lot of outcomes. 30% of kids in Uganda studies have sickle cell trait
- should acknowledge the different possible modifiers. gravidity, endemicity, sickle cell trait.
- Area with finite resources, may not be able to give it to every woman, but may be useful to give it to some.
- Other: infant anaemia as an adverse event of intrauteral exposure to malaria mention in paper under other morbidities
- Add physical development into box with neurocognitive development✓
- change colour in adult mortality add green✓
- reflect on different viewpoints of experts
- As a response to results from meeting one the presentation for meeting 2 was slightly adapted, in particular the exact wording of questions asked in the polls. To be absolutely clear it was understood the same way by everyone.

## Summary meeting 1

- Change triangle presence of P. Falciparum while pregnant to Pres of P. falciparum during pregnancy

1. Hypertension disorders of pregnancy
    - Keep in 4/4
    - split yes 3/4, no 1/4

    - might be best to just have eclampsia and pre-eclampsia which are biologically much more relevant than gestational hypertension, but data might be difficult to find, as often lumped together with much more prevalent outcome of gestational hypertension
2. Is there a causal link from maternal anaemia to clinical malaria
    - yes = keep arrow 1/4 , no = remove arrow 3/4
    - high risk of confounding for progression.
    - Difference between association and causal pathway
3. Morbidities
    - remove arrow and make into a shape like others. Discussed various options. Focused on oval shape.
    - Oval shape: yes 4/4
    - ensure it is clear that oval box of other morbidities is sequelae of the outcome that lead into it, but also potential any other morbidities that haven't been thought off yet or haven't been studied sufficiently yet
    - have some recognition in text about the nature of lifelong consequences of morbidities
    - less nephrons in fetus if mum exposed to MiP, huge, chronic kidney disease
    - arrow from child to other morbidities yes 4/4
    - arrow congenital malaria to other morbidities yes 4/4
    - arrow from fetal anaemia to other morbidities yes 4/4
4. Modified incidence of malaria in <5 to increased incidence of malaria in ,5
    - yes 4/4
5. fetal anaemia also to neurocongenital development impairment
    - fetal anaemia arrow to other morbidities and congenital malaria to other morbidities. 4/4. (actually not voted, 2 clearly stated, others nodded). Then congenital malaria to fetal anaemia, and fetal anaemia to neurocognitive development something to consider to add relationships, not really to incidence of malaria in <5 or immunity from fetal anaemia.

Other important comments:

Important outcome of this work is to identify areas that are low in data and share with research community.

Gravidity, Low and high endemicity are important factors to take account of for each of the outcomes in the model.

Probably not feasible at this stage but with foresight into the future to move away from a one size fits all model

association between sickle cell treat, seem to modify the risk in a lot of outcomes. 30% of kids in Uganda studies have sickle cell trait

should acknowledge the different possible modifiers. gravidity, endemicity, sickle cell trait.

Area with finite resources, may not be able to give it to every woman, but may be useful to give it to some.

As a response to results from meeting one the presentation for meeting 2 was slightly adapted, in particular the exact wording of questions asked in the polls. To be absolutely clear it was understood the same way by everyone.

## Summary meeting 2

1. Hypertension disorders of pregnancy
    - Keep in 5/5
    - split yes 1/5, no 4/5
    - reason for not splitting: difficult to get the right diagnosis. For example distinguish between severe malaria and eclampsia might be difficult.
    - Hypotension is linked to severe malaria  (a manifestation of it)
    - There is more hypertension disorder in pregnancy in Africa than was thought , while hypertension is always measured but often doesn't end up in the dataset.
    - should be there because of consequences, Case control study (4) Systematic review in Africa of hypertensive disorder and MiP 2.67 OR higher hypertensive disorder in women with MiP.
    - Likely that Africa has much higher rates of hypertension than Asia (also see African Americans for example).
    - Outcomes such as death and stillbirth/miscarriage same, but there are other outcomes from pre-eclampsia/eclampsia that are not captured, like mental health, stroke, other organ damage like liver, kidney, brain; also later in life heart disease, high blood pressure, kidney disease, stroke
2. Is there a causal link from maternal anaemia to clinical malaria
    - yes = keep arrow 1/5 , no = remove arrow 4/5
    - Rose said in addition to change her yes to a no.
3. Morbidities
    - Oval shape: yes 3/5 no 2/5
    - arrow from child to other morbidities yes 4/5 no 1/5
    - arrow congenital malaria to other morbidities yes 5/5
    - arrow from fetal anaemia to other morbidities yes 5/5
    - 
    - Paper should really highlight and discuss the potentially progressive nature of these morbidities
    - Ensure that it is clear that these other morbidities are not going to be quantified in an cost effectiveness paper, for the paper on the model it is really nice to have though.
    - Add green to mortality box
4. Modified incidence of malaria in <5 to increased incidence of malaria in ,5
    - yes 5/5

Other: infant anaemia as an adverse event of intrautral exposure to malaria mention in paper under other morbidities

Add physical development into box with neurocognitive development

change colour in adult mortality add green

reflect on different viewpoints of experts
