# Supplementary material for: Co-developing a comprehensive disease policy model with stakeholders: The case of malaria during pregnancy
Source: PLOS Glob Public Health. 2025 May 7;5(5):e0003775. doi: 10.1371/journal.pgph.0003775 (PMC12057981; doi:10.1371/journal.pgph.0003775)
Supplement: Attachment — Submitted filename: Response to reviewers_20250310.docx [file pgph.0003775.s008.docx]

**Reviewers' comments and response**

**Comments to the Author**

1. Does this manuscript meet PLOS Global Public Health’s [publication criteria](https://journals.plos.org/globalpublichealth/s/criteria-for-publication)? Is the manuscript technically sound, and do the data support the conclusions? The manuscript must describe methodologically and ethically rigorous research with conclusions that are appropriately drawn based on the data presented.

Reviewer #1: Yes

Reviewer #2: Yes

**Response**: N/A

2. Has the statistical analysis been performed appropriately and rigorously?

Reviewer #1: Yes

Reviewer #2: N/A

**Response:** N/A

3. Have the authors made all data underlying the findings in their manuscript fully available (please refer to the Data Availability Statement at the start of the manuscript PDF file)?

The [PLOS Data policy](https://journals.plos.org/globalpublichealth/s/data-availability) requires authors to make all data underlying the findings described in their manuscript fully available without restriction, with rare exception. The data should be provided as part of the manuscript or its supporting information, or deposited to a public repository. For example, in addition to summary statistics, the data points behind means, medians and variance measures should be available. If there are restrictions on publicly sharing data—e.g. participant privacy or use of data from a third party—those must be specified.

Reviewer #1: Yes

Reviewer #2: No

**Response:**

This study is a Delphi consultation study with twelve experts in the field of malaria during pregnancy or economics of malaria. Due to the small size of the Delphi panel (N=12), the detailed data by anonymised participant collected during the Delphi consultations cannot be made available as individuals participating in the Delphi panel would potentially be identifiable. However, a detailed analysis report, with all identifying information removed, have been submitted for both rounds of Delphi consultations as an appendix. All experts had agreed to be named as being an expert on the panel, however their answers have to be anonymised and remain confidential, hence the detailed data cannot be made available. For any further insights required beyond the summary reports, written representation can be made to the corresponding author.

4. Is the manuscript presented in an intelligible fashion and written in standard English?

PLOS Global Public Health does not copyedit accepted manuscripts, so the language in submitted articles must be clear, correct, and unambiguous. Any typographical or grammatical errors should be corrected at revision, so please note any specific errors here.

Reviewer #1: No

Reviewer #2: Yes

**Response:**

We have revised and edited the manuscript for typographical or grammatical errors.

5. Review Comments to the Author

Please use the space provided to explain your answers to the questions above. You may also include additional comments for the author, including concerns about dual publication, research ethics, or publication ethics. (Please upload your review as an attachment if it exceeds 20,000 characters)

**Reviewer #1:** The manuscript meets PLOS Global Public Health’s criteria because it demonstrates sound research methods, is ethically conducted, and presents findings that are well-supported by the data. The study’s conclusions are consistent with the data, and the methodology adheres to standards for rigor and reliability, making it suitable for publication. The statistical analysis in this study is both thorough and accurate, using suitable methods to examine the data, given that this paper is looking at using the Delphi technique and cost effectiveness. The authors apply the statistical techniques correctly, providing a solid basis for the conclusions drawn from the results. This level of rigor supports the reliability of the findings. The authors have fully complied with the PLOS data policy by making all relevant data accessible. They have either included the data in the manuscript or within the supplementary/supporting documents. This transparency enables reproducibility and further research, with the limitations properly disclosed and recommendations for further research also disclosed. They do also protect the names of the individuals who take part in the study (however I am not too sure if this is a must to disclose, thus I did not flag this as an issue).

The manuscript presents the research clearly, there are issues with language and grammar that could distort comprehension. The authors need to give a brief explanation to the concepts included in this paper, given that as a first time reader/someone who does not know much on malaria/pregnancy and their relationship, the concepts are not immediately clear. Improvements in grammar, phrasing, and consistency are recommended to ensure the language is as clear and precise as possible for readers. I would also recommend getting an editor to edit the final piece of work, given the grammatical errors in the paper. While there are not too many, the authors would benefit from this greatly.

Response:

Thank you for your thorough review of this paper and positive feedback. We are encouraged that you recognized the importance of this research, as well as our efforts in conducting it with appropriate methods and presenting the work in a rigorous way.

We have edited the text and trust that we have removed any errors and improved comprehension.

Regarding the concepts of malaria during pregnancy and its relationship with other outcomes, we have added the following sentences (shown in tracked changes) to help readers with little or no experience in malaria during pregnancy and/or cost-effectiveness analysis:

“Because health interventions can have multiple health effects (for example, both morbidity and mortality), composite metrics such as Quality Adjusted Life Years (QALYs) or Disability Adjusted Life Years (DALYs) are frequently used. Complexity is increased when a disease affects more than one population group.

Malaria during pregnancy has been shown to be associated with a wide range of outcomes affecting both, the mother and the child. Outcomes in the pregnant woman include clinical malaria, anaemia and increased maternal mortality. In the child, malaria during pregnancy can lead to, amongst other outcomes, stillbirth, low birth weight and increased <5 mortality. (1). In this paper, we use malaria in pregnancy as a case study to illustrate how a structured approach to co-developing a disease policy model with relevant stakeholders can result in a more robust model, which will carry greater influence with the scientific and policy community because of the multi-disciplinary input into its development. “

**Reviewer #2:** Thank you for the opportunity to review your article. This is a compelling and insightful contribution to an important area of research. The article is well-structured, and the inclusion of tables significantly enhances clarity by aligning effectively with the written discussion. The tables provide a clear depiction of the methodological steps undertaken in the Delphi process, thereby strengthening the overall coherence and comprehensibility of the study.

**Response:**

Thank you for reviewing our paper and for providing us with such encouraging and positive feedback. We are very glad that you recognize the importance and significance of this research.

6. PLOS authors have the option to publish the peer review history of their article ([what does this mean?](https://journals.plos.org/globalpublichealth/s/editorial-and-peer-review-process#loc-peer-review-history)). If published, this will include your full peer review and any attached files.


**Do you want your identity to be public for this peer review?** If you choose “no”, your identity will remain anonymous but your review may still be made public.


For information about this choice, including consent withdrawal, please see our [Privacy Policy](https://www.plos.org/privacy-policy).

Reviewer #1: No

Reviewer #2: No

[NOTE: If reviewer comments were submitted as an attachment file, they will be attached to this email and accessible via the submission site. Please log into your account, locate the manuscript record, and check for the action link "View Attachments". If this link does not appear, there are no attachment files.]

While revising your submission, please upload your figure files to the Preflight Analysis and Conversion Engine (PACE) digital diagnostic tool, https://pacev2.apexcovantage.com/. PACE helps ensure that figures meet PLOS requirements. To use PACE, you must first register as a user. Registration is free. Then, login and navigate to the UPLOAD tab, where you will find detailed instructions on how to use the tool. If you encounter any issues or have any questions when using PACE, please email PLOS at figures@plos.org. Please note that Supporting Information files do not need this step.
